# Associations between the gut microbiota at one-year and neurodevelopment in children from the SEPAGES cohort

**DOI:** 10.1016/j.bbih.2025.101063

**Published:** 2025-07-18

**Authors:** Aline Davias, Sarah Lyon-Caen, Nina Iszatt, Celine Monot, Yamina Rayah, Zehra Esra Ilhan, Karine Guichardet, Sam Bayat, Séverine Valmary-Degano, Gina Muckle, Merete Eggesbø, Patricia Lepage, Claire Philippat, Rémy Slama

**Affiliations:** a Environmental Epidemiology applied to Development and Respiratory Health team, Institute for Advanced Biosciences, University Grenoble Alpes, Inserm, CNRS, Grenoble, France; b Division of Climate and Environmental Health, Norwegian Institute of Public Health (NIPH), 0213, Oslo, Norway; c Université Paris-Saclay, INRAE, AgroParisTech, Micalis Institute, F-78350, Jouy-en-Josas, France; d Pediatric Department, Grenoble Alpes University Hospital, 38700, Grenoble, France; e Pulmonology and Physiology Department, Grenoble Alpes University Hospital, 38700, Grenoble, France; f Biobank BB-0033-00069, Univ. Grenoble Alpes, Inserm U1209, CNRS UMR5309, Institute for Advanced Biosciences, CHU Grenoble-Alpes, F-38000, Grenoble, France; g Centre Hospitalier Universitaire de Québec Research Centre and School of Psychology, Laval University, Québec City, Québec, Canada; h Department of Clinical and Molecular Medicine, NTNU, Trondheim, Norway; i Faculty of Veterinary Medicine, Norwegian University of Life Sciences, 1432, Ås, Norway; j SMILE, Institut de Biologie de l'ENS (IBENS), Ecole Normale Supérieure, Université PSL, CNRS, INSERM, F-75005, Paris, France; k PARSEC, Ecole Normale Supérieure, Université PSL, CNRS, INSERM, F-75005, Paris, France

**Keywords:** Child behavior, Child cognition, Couple-child cohort, Gut microbiota, Neurodevelopment

## Abstract

Fundamental research indicates a communication between the gut microbiota and the central nervous system, referred to as the microbiota-gut-brain axis. This link is little characterized in humans in the general population. We prospectively investigated the relationships between the gut microbiota composition of one-year-old children and neurodevelopment parameters at 2 and 3 years of age. Within the SEPAGES French couple-child cohort, we profiled gut microbiota by sequencing the V3-V4 region of the 16S rRNA gene from stool samples in 356 children at 12 months of age. We later assessed children's neurodevelopment through validated tests (CBCL at 2 years, and SRS-2, BRIEF-P and WPPSI-IV at 3 years). Microbial α-diversity indices, the 4 most abundant phyla, and the 46 most abundant genera were analyzed for their relations with neurodevelopmental parameters using multiple linear regression, while associations of β-diversity with neurodevelopment were examined through PERMANOVA tests. α- and β-diversity indices were not associated with neurodevelopmental parameters in children. Suggestive associations were observed with taxonomy, but not maintained after correction for multiple comparisons: phyla Proteobacteria and Bacteroidetes tended to be associated with higher socio-emotional neurodevelopment assessed with different sub-scores; phylum Firmicutes with increased plan and organization problems; genera *Lactococcus*, *Coprococcus, Oscillibacter, Clostridium XVIII*, *Veillonella, Parabacteroides, Subdoligranulum* and *Saccharibacteria genera incertae sedis* with lower socio-emotional neurodevelopment, while genera *Enterococcus* and *Butyricicoccus* tended to be associated with higher socio-emotional neurodevelopment, assessed with different sub-scores. Within this generally healthy population, only suggestive associations were observed between gut microbiota composition and neurodevelopmental scores at 2 and 3 years. Larger studies are needed to examine a possibly weak link between the gut microbiota of one-year-old children and their neurodevelopment.

## Highlights

• We investigated the link between infant gut microbiota and neurodevelopmental scores.
• α- and β-diversity indices were not associated with neurodevelopmental scores.
• Suggestive associations of gut microbiota with neurodevelopmental scores were seen.
• These associations were not maintained after correction for multiple comparisons.
• Larger prospective studies are needed to examine a possibly weak link.

## 1 Introduction

Neurodevelopment refers to the growth and development of the central nervous system from conception through adulthood (Stiles and Jernigan, 2010). Proper neurodevelopment is essential for cognitive and behavioral functioning, and disruptions in this process can lead to neurodevelopmental disorders such as autism spectrum disorders (ASD) or attention-deficit/hyperactivity disorder (ADHD) that result in impairments of personal, social, academic, or occupational functioning, according to the Diagnostic and Statistical Manual of Mental Disorders, Fifth Edition (American Psychiatric Association, 2013). Their pathophysiology are not well understood, leading to treatments targeting symptoms rather than underlying causes (Masi et al., 2017). Therapies, apart from early-life behavioral interventions, are often limited (Dawson and Burner, 2011; McPheeters et al., 2011).

The microbiota-gut-brain axis (Cryan et al., 2019; Wang et al., 2023) is defined as a bidirectional homeostatic route of communication between the gut microbiota, which consists of trillions of microorganisms residing in the lumen of the gastrointestinal tract, the gastrointestinal tract containing the enteric nervous system (ENS), and the central nervous system (CNS) through immune (Dantzer et al., 2008; Duerkop et al., 2009; Forsythe and Bienenstock, 2010; Sternberg, 2006), metabolic (Gundersen and Blendy, 2009; MacFabe et al., 2011; Nicholson et al., 2012; Thomas et al., 2012) and neuronal signaling pathways (Barrett et al., 2012; Forsythe and Kunze, 2013; Lyte, 2011).

The first years of life represent a critical window for neurodevelopment, during which key processes such as synaptogenesis, myelination, and the refinement of neural circuits supporting cognition, language, and socio-emotional behavior are progressing (Fox et al., 2010). During this period, disruptions in gut microbiota composition have been hypothesized to influence neurodevelopment via immune modulation, microbial metabolite production (e.g., short-chain fatty acids, neurotransmitter precursors), and regulation of the hypothalamic-pituitary-adrenal (HPA) axis, which collectively contribute to brain maturation and function (Cryan et al., 2019). Experimental animal models support a potential link between gut microbiota and neurodevelopment through the microbiota-gut-brain axis. Germ-free mice showed phenotypic traits consistent with autism, including deficits in social interaction, which can be reversed by gut colonization after weaning (Desbonnet et al., 2014). Several epidemiological case-control studies have compared the gut microbiota in children diagnosed with ASD (Ahmed et al., 2020; Averina et al., 2020; Chen et al., 2020; Dan et al., 2020; De Angelis et al., 2013; Ding et al., 2020, 2021; Ha et al., 2021; Inoue et al., 2016; Kang et al., 2013, 2018; Kushak et al., 2017; Li et al., 2019; Liu et al., 2019; Ma et al., 2019; Plaza-Díaz et al., 2019; Pulikkan et al., 2018; Rose et al., 2018; Strati et al., 2017; Sun et al., 2019; Tomova et al., 2020; Zhai et al., 2019; Zhang et al., 2018; Zou et al., 2020; Zurita et al., 2020) or ADHD (Aarts et al., 2017; Jiang et al., 2018; Prehn-Kristensen et al., 2018; Richarte et al., 2021; Szopinska-Tokov et al., 2020; Wan et al., 2020; Wang et al., 2020, 2022) to neurotypical siblings or community controls and observed notable differences. However, these retrospective studies are potentially subject to reverse causation, not allowing to identify the direction of any causal link; moreover, they do not indicate if alterations of the gut microbiota could lead to mild neurodevelopmental variations, such as those observed in the general population.

A few prospective studies have been conducted in the general population on the link between gut microbiota and neurodevelopmental scores (Aatsinki et al., 2019; Carlson et al., 2018; Laue et al., 2020; Loughman et al., 2020; Sordillo et al., 2019; Tamana et al., 2021). In a prospective study of 405 infants, Tamana et *al.* found that infants with an enriched abundance of *Bacteroides* in their gut microbiota at 1 year had higher cognitive and language scores indicative of greater development at the age of 2, using the Bayley Scales Of Infant and Toddler Development (BSID-III) (Tamana et al., 2021). Similarly, in a cohort study of 89 infants, Carlson et *al.* found enrichment of gut microbiota with *Bacteroides* genus at 12 months to be associated with higher cognitive development scores using the Mullen scale at 2 years of age (Carlson et al., 2018). In an Australian birth-cohort study of 201 children, Loughman et *al.* found an association between decreased abundance of genus *Prevotella*, another taxon in the Bacteroidetes phylum, in gut microbiota at 12 months, and increased behavioral problems at 2 years, in particular, internalizing problems, using the CBCL score (Loughman et al., 2020). On the other hand, in a study of 309 children, Sordillo et al. found that a *Bacteroides*-dominant gut microbiota at 6 months was associated with poorer fine motor scores and increased odds for potential delay in fine motor skills at 3 years using the Ages and Stages Questionnaire, third edition (ASQ-3), and abundance of *Ruminococcus*, a genus in the *Lachnospiraceae* family, was related to poorer ASQ-3 communication and personal/social scores (Sordillo et al., 2019). Laue et *al*. found that several gut microbiota taxa at 1, 2 and 3 years, including many in the *Lachnospiraceae* family, were associated with social behaviors at age three using the Social Responsiveness Scale (SRS-2) (Laue et al., 2020). Finally, Aatsinki et *al.* study on 301 children from the FinnBrain Birth Cohort suggested that gut microbiota composition at 2.5 months was related to infant temperament at six months assessed by maternal report using the Infant Behavior Questionnaire-Revised (IBQ-R): more *Bifidobacterium* and *Enterobacteriaceae* was associated with better behavior regulation, higher abundance of *Bifidobacterium* and *Streptococcus* was associated with positive emotionality, and greater diversity was associated with lower negative emotionality and fear reactivity (Aatsinki et al., 2019).

All in all, studies describing a microbiota-gut-brain axis in animal models are numerous, some supporting the involvement of this axis in neurodevelopment. Relatively few prospective epidemiological studies have investigated this possible link between gut microbiota and neurodevelopment in humans, underscoring the necessity for further investigation using diverse and larger cohorts. This study aims to investigate the relationship between gut microbiota at one year and neurodevelopmental parameters at two and three years of age.

## 2 Materials and methods

### 2.1 Design of SEPAGES couple-child cohort

We used the data from the French couple-child cohort SEPAGES (Suivi de l’Exposition à la Pollution Atmosphérique durant la Grossesse et Effet sur la Santé) (Lyon-Caen et al., 2019). The SEPAGES cohort participants recruited between 2014 and 2017 were pregnant women, their partner and future children, living approximately in a buffer of 80 km around the center of Grenoble. Pregnant women met eligibility criteria such as: being pregnant by less than 19 gestational weeks at inclusion, being older than 18 years, having a singleton pregnancy, living in the study area and planning to give birth in one of the four maternity clinics from Grenoble area in France. From the 484 families included in SEPAGES cohort, the sample size with full gut microbiota and neurodevelopment data in our study was between 310 and 338 (depending on neurodevelopmental parameters availability). All required ethical and data privacy agreements were obtained and all enrolled families signed an informed consent form. Details of the SEPAGES cohort are available elsewhere (Lyon-Caen et al., 2019).

### 2.2 Assessment of the gut microbiota

Details of the methods used to assess the gut microbiota at one year in SEPAGES cohort are described in Davias et al. (2024). Briefly, parents from SEPAGES cohort were asked to collect fecal samples of their offspring in a sealed container at approximately one year (median age of 54 weeks), a sensitive period when the gut microbiota undergoes rapid development (Fleming et al., 2018; Poston, 2012; Suzuki, 2018). Samples were immediately stored at −20 °C at the volunteer's home for up to one week, then transported on ice to the biobank where they were separated into three aliquots and stored at −80 °C. Fecal samples were aliquoted from 360 children at one year (∼150 mg). Total DNA was extracted from all samples with the PowerFecal DNA Isolation Kit (Qiagen), with a prior homogenization including chemical and mechanical lysis steps. The V3-V4 hypervariable region of the 16S ribosomal ribonucleic acid (rRNA) gene was amplified (Klindworth et al., 2013). Following quality control and sequencing library construction, high-throughput sequencing was performed (MiSeq Illumina). Reads were further corrected for known sequencing errors using SPAdes (Bankevich et al., 2012) and then merged using PEAR (Zhang et al., 2014). Amplicon sequencing variants (ASV) were identified using a Vsearch pipeline (Rognes et al., 2016). ASV taxonomical classification was performed using a classifier from the RDPTools suit (Cole et al., 2014). From the 360 stool samples at one year, we removed 4 samples which had a low DNA or sequencing quality (samples with number of reads <2500). To control for variation in sequencing depth for diversity analyses, the ASV matrix was rarefied to 5000 sequences, which led to the removal of 6 additional samples (n = 350).

Estimates of α-diversity (i.e. diversity within a sample) were calculated using the specific richness and the Shannon diversity indices, on the rarefied ASVs Table (5,000 sequences) with R program version 4.1.0, package Phyloseq version 1.27.6 (McMurdie and Holmes, 2013). The specific richness index is the number of different ASVs observed within a fecal sample. The Shannon diversity index was defined as ${\left(H\right)=-\sum_{i=1}^{s}p_{i}\;\log\;p}_{i}$ with $p_{i}=\frac{n_{i}}{N}$ , *n*_*i*_ is the number of bacteria found for a particular ASV _*i*_, *N* is the total number of bacteria found in the sample, and *s* is the total number of different ASV in the sample (Shannon and Weaver, 1964). β-diversity between each given pair of gut microbiota samples was calculated using the Bray-Curtis dissimilarity metric, defined on the rarefied ASVs Table (5,000 sequences), as ${BC}_{ij}=1-\left(2C_{ij}\right)/\left(S_{i}+S_{j}\right)$ where *C*_*ij*_ is the sum of the lower values for the ASV found in each sample and *S*_*i*_ and *S*_*j*_ are the total number of bacteria counted in samples i and j respectively (Bray and Curtis, 1957).

Taxonomic affiliation was described at all taxa levels. Null values in genera variables were replaced by the value 1/5000 before the variables were log-transformed. The relative abundances of the 4 most abundant phyla and the 46 most abundant genera (detected in at least 30 % of the children at one year) were considered in the analyses.

All gut microbiota variables were standardized for technical factors (child age at stool collection and MiSeq analytic batch effect) if they were associated with a p-value<0.2.

### 2.3 Assessment of neurodevelopment

Socio-emotional and cognitive dimensions of neurodevelopment as defined by Forns (Forns et al., 2012) were evaluated in children at 2 and 3 years of age using questionnaires completed by the parents (Child Behavior Checklist 1.5–5 (CBCL) (Achenbach, T.M. and Rescorla, L., 2000), Behavior Rating Inventory of Executive Function, Preschool Version (BRIEF-P) (Gioia et al., 2003), Social Responsiveness Scale, Second Edition (SRS-2) (Constantino and Gruber, 2012)) and tests administered to children by neuropsychologists (French version of the Wechsler Preschool and Primary Scale of Intelligence, 4th edition (WPPSI-IV) (Wechsler, 2014; O’Donnell, 2009)). This scale was translated, culturally adapted to French norms, and calibrated in a representative sample of French children aged between 2.5 and 7.25 years by the Editions of the Center in Applied Psychology (O'Donnell, 2014).

The CBCL (Achenbach, T.M. and Rescorla, L., 2000) was completed by one of the parents when the child was two years old. We focused on the externalizing (the sum of the attention and aggressive scores) and internalizing (the sum of the emotionally reactive, anxious/depressed, somatic complaints and withdrawn scores) scores. Parents completed the SRS-2 and the BRIEF-P when the child was aged 3 years. The SRS-2 is a 65-item questionnaire recognized as a valid and reliable quantitative measure of child social behavior and autistic traits in both population and clinical settings (Constantino and Gruber, 2012). The SRS-2 is correlated (0.7) with the Autism Diagnostic Interview-Revised (ADI-R), a gold standard for research diagnosis of ASD (Constantino et al., 2003). We focused on the total SRS-2 score (addition of 5 scores: social awareness, social cognition, social communication, social motivation, and restricted interests and repetitive behaviors). The BRIEF-P is a 63-item questionnaire within 5 scores that measure different aspects of executive functioning: inhibit, shift, emotional control, working memory, and plan/organize (Gioia et al., 2003). WPPSI-IV test, which was administered to children at 3 years of age by trained neuropsychologists, provided scores of Intelligence Quotient (IQ): verbal comprehension, visual-spatial, and working memory and a total score (Wechsler, 2014; (O’Donnell, 2009), 2014).

WPPSI-IV scores were continuous scores standardized on child age at assessment and on the neuropsychologist who conducted the test. For all other neurodevelopmental parameters, continuous raw scores were used. Higher CBCL and SRS-2 scores indicate higher socio-emotional/behavioral impairments; higher BRIEF-P scores indicate higher cognitive disabilities whereas higher WPPSI-IV scores indicate greater cognitive function.

### 2.4 Statistical analyses

The parameters of child neurodevelopment considered in relation to gut microbiota parameters were the 2 CBCL behavioral scores, the SRS-2 total social behavior score, the 5 BRIEF-P executive function scores, the 4 WPPSI-IV cognitive function scores.

#### 2.4.1 Covariates

We used a directed acyclic graph (DAG) to describe causal relations and identify *a priori* the covariates to be included in the analyses (Fig. S1). All analyses were adjusted for: child age at neurodevelopment assessment (continuous), except for WPPSI-IV scores, that were already age-standardized, delivery mode (vaginal delivery *vs*. Cesarian section), gestational duration (continuous), child characteristics (sex (male *vs*. female), birthweight (<3 kg, 3–3.4 kg, ≥3.5 kg) and birth length (<50 cm, 50–51 cm, ≥52 cm)), breastfeeding duration (not breastfed, <24 weeks, 24–47 weeks, still breastfeed at 48 weeks), period of introduction of solid food (between 0 and 6 months old, between 6 and 12 months old, not introduced at 12 months old), antibiotics use during the first year of life (no *vs*. yes), presence of pets at home (no *vs*. one or more), child perinatal passive smoking up to one year (no *vs*. yes), quality of cognitive stimulation and emotional support in the child's home environment at 3 years (Home Observation Measurement of the Environment (HOME) questionnaire total score, continuous), main mode of child care at 12 months (collective day care *vs*. other) and maternal characteristics (parity (none *vs*. one child or more), age (continuous), body mass index (BMI) before pregnancy (<19 kg/m^2^, 19–23.9 kg/m^2^, ≥24 kg/m^2^), education (≥5 years *vs*. <5 years after graduation from high school), smoking status during pregnancy (no *vs*. yes), and maternal anxiety and depression score during the third trimester of pregnancy (continuous)). We imputed missing values of adjustment covariates using simple imputation by predictive mean matching for continuous covariates, logistic regression for binary covariates and polytomous logistic regression for covariates with 3 or more categories (Little, 1988; Rubin, 1986).

#### 2.4.2 Association between α- and β-diversity and child neurodevelopment

For α-diversity (specific richness and Shannon diversity indices), we used multiple linear regression models (one model per α-diversity index and neurodevelopmental score) adjusted for the above-mentioned covariates. For β-diversity, we investigated group differences in Bray Curtis β-diversity for low (1st tertile), medium (2nd tertile), and high (3rd tertile) neurodevelopment scores by testing significance of pairwise groups of exposure with Permutational Multivariate Analysis of Variance Using Distance Matrices (PERMANOVA), with R program version 4.2.2, package Vegan version 2.6–4, Adonis2 function (Anderson, 2001). β-diversity analyses were adjusted for the same covariates as the α-diversity analyses.

To limit associations due to multiple comparisons, a family-wise error rate (FWER) correction to the p-value threshold was applied on the p-values of the α- and β-diversity analyses. The correction uses a Bonferroni procedure extended to handle correlated tests: the actual number of associations being tested (T) is replaced by the effective number of independent tests (Te). Te is estimated by the effective number of independent explanatory variables $\sum_{i=1}^{M}\left[I_{\left(\lambda_{i}>1\right)}x\left(\lambda_{i}-1\right)\right]$ multiplied by the effective number of independent outcomes $\sum_{i=1}^{N}\left[I_{\left(\lambda_{i}>1\right)}x\left(\lambda_{i}-1\right)\right]$, where I(x) is an indicator function and λ_i_ were the eigenvalues of the matrix of correlations between M explanatory variables and the eigenvalues of the matrix of correlations between N outcomes. The p-value threshold to control FWER to α (in our case, 5 %), using Te in a Bonferroni procedure, is then α/Te (adapted from Li et al. (2012)). The 3 diversity indices corresponded to 3 independent predictors and the 12 neurodevelopmental parameters to 7 independent outcomes. The corrected significance level was thus 0.05/(3x7) = 0.002. Coherently with good epidemiological practices, we did not hide the non-significant associations, to allow future confirmatory studies or meta-analyses.

#### 2.4.3 Association between taxonomy and child neurodevelopment

For taxonomy analyses, we applied multiple linear regression models (one model per taxon and neurodevelopmental score) using the 4 most abundant phyla and the 46 most abundant genera (detected in at least 30 % of the children) as explanatory variables. These analyses were adjusted for the same covariates as the diversity analysis. Taxa counts were converted to relative abundances. We applied a FWER multiple comparison correction for all associations between bacterial taxa and neurodevelopmental parameters. The 50 taxa variables corresponded to 33 independent predictors and the 12 neurodevelopmental parameters to 7 independent outcomes. The corrected significance level was thus 0.05/(33x7) = 0.0002.

Finally, for each neurodevelopmental parameter, we performed a multiple linear regression including all gut microbiota parameters that were associated with this specific outcome in the previous regression models, adjusting for the same covariates. We examined Cohen's f^2^ to compare the R^2^ of these models with the R^2^ of the models that included only the covariates to understand the extent to which these identified gut microbiota parameters contributed to explaining the variance in the neurodevelopmental parameters. Cohen's f^2^ is used to assess the effect size, with values of 0.10, 0.25, and 0.40 typically corresponding to small, medium, and large effect sizes, respectively (Cohen, 1988).

#### 2.4.4 Sensitivity analyses

As sensitivity analyses, we repeated the α-diversity analyses using two different thresholds of rarefaction (5,000 and 10,000 reads). We repeated the analyses for CBCL scores without the HOME score as this covariate, used as a proxy at 2 years of the cognitive stimulation and emotional support in the child's home environment in the main analyses, was assessed at 3 years and thus after the CBCL assessment. We reproduced the WPPSI-IV analyses without standardizing for the neuropsychologist who conducted the test. We reproduced all the analyses without standardizing for technical factors (age of child at stool collection and MiSeq batch effect) of gut microbiota parameters and without adjusting on predictors of gut microbiota that were not related to neurodevelopment in our DAG (delivery mode, antibiotics use during the first year of life and presence of pets at home). Finally, we investigated potential non-monotonic relations between gut microbiota parameters (the 2 α-diversity indices, the 4 phyla and the 46 genera) and neurodevelopmental parameters: we conducted heterogeneity tests in outcome value across gut microbiota parameters tertiles and trend tests using continuous variables whose values corresponded to the tertile specific median gut microbiota parameter levels. Associations in which gut microbiota parameters exhibited heterogeneity in outcome across tertiles but with little support for a consistent pattern (indicated by a low p-value in the heterogeneity test but a high p-value in the trend test) were considered as suggestive of a non-monotonic association.

All statistical analyses were performed with R program version 4.2.2.

## 3 Results

### 3.1 Study population

Out of 484 families participating in SEPAGES cohort, 356 had available child gut microbiota data at one year. Women included in our analyses were highly educated (57 % had 5 years of studies or more after graduation from high school) and 94 % of them did not smoke during pregnancy. Most of them (63 %) had a BMI between 19 and 24 kg/m^2^; 84 % of the mothers gave birth vaginally. No significant difference existed between the women included in our analyses and those excluded (Table 1). The proportion of included children with a weight at birth under 3 kg was 21 %; 8.4 % of children were not breastfed at birth, while 23 % were still breastfed at 48 weeks. Children included in our analyses were more frequently exposed to antibiotics during the first year of life compared to those excluded (p-value: 0.02, Table 1). Distribution of gut microbiota parameters and neurodevelopmental scores in the SEPAGES cohort are available in Table S1and Table S2, respectively. Pearson's correlations between neurodevelopmental parameters are available in Fig. S2. Briefly, the WPPSI-IV scores were not correlated with any of the other scores, while moderate correlations (r ≤ 0.8) were observed across SRS-2, CBCL and BRIEF-P scores.

**Table 1:** Description of pregnant women and their child from SEPAGES cohort included in the study, compared to the rest of the SEPAGES cohort (484 mother-child pairs).

Maternal and child characteristics	Included in the analyses			Excluded from our analyses	p-value^b^
	N^a^	n (%); Median (IQR)		n (%); Median (IQR)	
**Maternal education**	354				0.80
<5 years after graduation		153 (43 %)		57 (45 %)	
≥5 years after graduation		201 (57 %)		71 (55 %)	
**Maternal age at conception (years)**	356	32 (30, 35)		32 (30, 35)	0.29
**Maternal BMI before pregnancy**	355				0.40
<19 kg/m^2^		48 (14 %)		12 (9.6 %)	
19–23.9 kg/m^2^		223 (63 %)		78 (62 %)	
≥24 kg/m^2^		84 (24 %)		35 (28 %)	
**Maternal anxiety and depression score during the third trimester of pregnancy**	342	10 (7, 13)		11 (7, 15)	0.16
**Maternal active smoking during pregnancy**	330				0.31
No		310 (94 %)		91 (91 %)	
Yes		20 (6.1 %)		9 (9.0 %)	
**Maternal parity**	356				0.88
None		164 (46 %)		58 (45 %)	
1 child or more		192 (54 %)		70 (55 %)	
**Gestational duration, completed weeks**	356	40 (39, 40)		39 (38, 40)	0.31
**Delivery mode**	356				0.54
Vaginal delivery		298 (84 %)		100 (81 %)	
Cesarean section		58 (16 %)		23 (19 %)	
**Child sex**	356				0.96
Male		190 (53 %)		66 (54 %)	
Female		166 (47 %)		57 (46 %)	
**Child birth weight**	356				0.96
<3 kg		74 (21 %)		25 (20 %)	
3–3.4 kg		174 (49 %)		62 (50 %)	
≥3.5 kg		108 (30 %)		36 (29 %)	
**Child birth length**	355				0.44
<50 cm		130 (37 %)		40 (34 %)	
50–51 cm		141 (40 %)		54 (46 %)	
≥52 cm		84 (24 %)		23 (20 %)	
**Breastfeeding duration**	347				0.54
Not breastfed		29 (8.4 %)		11 (12 %)	
<24 weeks		122 (35 %)		35 (37 %)	
24–47 weeks		117 (34 %)		32 (34 %)	
Still breastfeed at 48 weeks		79 (23 %)		16 (17 %)	
**Period of introduction of solid food**	319				0.09
Between 0 and 6 months of age		253 (79 %)		40 (67 %)	
Between 6 and 12 months of age		50 (16 %)		16 (27 %)	
Not introduced at 12 months		16 (5.0 %)		4 (6.7 %)	
**Presence of pets at home**	311				0.30
No		222 (71 %)		49 (65 %)	
One or more		89 (29 %)		26 (35 %)	
**Child antibiotics use between 0**–**12 months of age**	355				0.02
No		246 (69 %)		86 (81 %)	
Yes		109 (31 %)		20 (19 %)	
**HOME questionnaire total score**^c^	313	25 (23, 26)		25 (23, 27)	0.68
**Child perinatal passive exposure to smoking up to one year**	344				0.08
No		232 (67 %)		45 (57 %)	
Yes		112 (33 %)		34 (43 %)	
**Main mode of childcare at 12 months**	331				0.28
Collective day care		271 (82 %)		55 (76 %)	
Other		60 (18 %)		17 (24 %)	
**Child age at the CBCL**^d^**assessment (weeks)**	336	106 (105, 108)		106 (105, 107)	0.48
**Child age at the SRS-2**^d^**and BRIEF-P**^d^**assessment (weeks)**	341	158 (156, 160)		159 (157, 163)	0.002

a Number of observations before simple imputation of the missing values by predictive mean matching for continuous covariates, logistic regression for binary variables and polytomous logistic regression for covariates with 3 or more categories.

b Pearson's Chi-squared test; Wilcoxon rank sum test; Fisher's exact test.

c Home Observation Measurement of the Environment questionnaire.

d CBCL: Child Behavior Checklist, SRS-2: Social Responsiveness Scale and BRIEF-P: Behavior Rating Inventory of Executive Function, Pre-school.

### 3.2 Relation between maternal and child characteristics and child neurodevelopment

Adjusted associations between maternal and child characteristics and neurodevelopment are available in Table S3. Maternal education under 5 years after graduation was associated with higher child social behavior problem scores (total SRS-2 score) and executive functions (plan and organization BRIEF-P score), compared to maternal education higher or equal to 5 years after graduation. Children of multiparous mothers had lower scores of behavioral problems (internalizing CBCL score, SRS-2 total score and all BRIEF-P scores) and a higher work memory WPPSI-IV score, compared to children of primiparous mothers. Longer breastfeeding duration was associated with higher WPPSI-IV scores of verbal comprehension, work memory and total IQ. A higher HOME score at 3 years was associated with lower CBCL and SRS-2 scores of behavioral problems, and higher WPPSI-IV scores of verbal comprehension, work memory and total IQ. Higher maternal anxiety and depression score during the third trimester of pregnancy was associated with higher scores of behavior problems (CBCL scores and SRS-2 total score) and executive functions (all BRIEF-P scores). Collective childcare at 12 months was associated with lower BRIEF-P shift problem score and higher WPPSI-IV total IQ score, compared to other main modes of childcare. Finally, child perinatal exposure to passive smoking up to one year was associated with higher CBCL internalizing problems score.

### 3.3 Relation between gut microbiota diversity and child neurodevelopment

The number of children included in the diversity analyses varied between 310 and 333, depending on the availability of neurodevelopment scores. There was no strong evidence of an association between any of the α-diversity indices assessed from the children's gut microbiota at 1 year of age, and the children's neurodevelopmental scores at 2 or 3 years of age, in multiple linear regressions adjusted for *a priori* chosen covariates (lowest p-value, 0.06 for a statistical significance threshold corrected for multiple testing of p = 0.002; Table 2). Cohen's f^2^ comparing the R^2^ of the model with the α-diversity index and the covariates as explanatory variables to the R^2^ of the model with only the covariates as explanatory variables were all small (Cohen's f^2^ < 0.1, Table 2). There was no evidence of variations of Bray-Curtis β-diversity across neurodevelopment score tertile groups (Table 3).

**Table 2:** Adjusted associations between α-diversity indices of the child gut microbiota at one year of age and neurodevelopmental scores at two and three years in the SEPAGES cohort (sample sizes between 310 and 333).

Estimated effect of α-diversity indices on neurodevelopmental scores^a^	N^b^	Beta^c^	95 % CI^d^	p-value^e^
**CBCL assessed at 2 years**				
**Internalizing score**	332			
Specific richness		0.07	−0.13, 0.27	0.49
Shannon diversity		0.41	−0.53, 1.34	0.39
**Externalizing score**	331			
Specific richness		−0.04	−0.31, 0.22	0.75
Shannon diversity		0.57	−0.69, 1.82	0.37

**SRS-2 assessed at 3 years**				
**Total score**	327			
Specific richness		0.21	−0.32, 0.74	0.44
Shannon diversity		0.15	−2.34, 2.64	0.91

**BRIEF-P assessed at 3 years**				
**Inhibition score**	325			
Specific richness		0.07	−0.15, 0.29	0.51
Shannon diversity		0.15	−0.89, 1.19	0.78
**Shift score**	333			
Specific richness		0.03	−0.10, 0.15	0.69
Shannon diversity		−0.13	−0.72, 0.46	0.67
**Emotional control score**	332			
Specific richness		0.14	−0.01, 0.28	0.06
Shannon diversity		0.55	−0.14, 1.23	0.12
**Work memory score**	324			
Specific richness		0.09	−0.10, 0.27	0.35
Shannon diversity		0.10	−0.77, 0.98	0.81
**Plan and organization score**	330			
Specific richness		0.09	−0.03, 0.21	0.12
Shannon diversity		0.22	−0.34, 0.77	0.44

**WPPSI-IV assessed at 3 years**				
**Verbal comprehension score**	310			
Specific richness		−0.14	−0.63, 0.35	0.57
Shannon diversity		−1.49	−3.85, 0.88	0.22
**Visuospatial score**	310			
Specific richness		−0.20	−0.68, 0.27	0.40
Shannon diversity		−0.31	−2.59, 1.96	0.79
**Work memory score**	310			
Specific richness		−0.10	−0.57, 0.36	0.66
Shannon diversity		−1.47	−3.71, 0.76	0.20
**Total score**	310			
Specific richness		−0.14	−0.62, 0.34	0.56
Shannon diversity		−1.24	−3.55, 1.07	0.29

All Cohen's f values were lower than 0.1.

a Child Behavior Checklist (CBCL), Social Responsiveness Scale (SRS-2) and Behavior Rating Inventory of Executive Function, Pre-school (BRIEF-P) are continuous raw scores. Wechsler Preschool and Primary Scale of Intelligence (WPPSI-IV) are continuous scores standardized on child age at evaluation and on the neuropsychologist who conducted the test.

b Number of observations.

c Average changes in neurodevelopmental scores when the specific richness increased by 10 ASVs or when the Shannon diversity increased by one unit; adjusted for child age at neurodevelopment assessment (except for WPPSI-IV scores that were already standardized), delivery mode, gestational duration, child characteristics (sex, weight and length at birth), breastfeeding duration, period of introduction of solid food, antibiotics use during the first year of life, presence of pets at home, maternal parity, child perinatal passive smoking up to one year, HOME questionnaire total score, main mode of child care at 12 months and maternal characteristics (parity, age and BMI before pregnancy, education, and smoking status during pregnancy, maternal anxiety and depression score during the third trimester of pregnancy).

d CI: Confidence Interval.

e After correction for multiple testing, the significance threshold was 0.002.

**Table 3:** Adjusted associations between β-diversity of the child gut microbiota at one year of age and the neurodevelopment at two and three years in the SEPAGES cohort (sample sizes between 310 and 333).

Neurodevelopmental scores^a^	N^b^	Sum of Squares^c^	F-statistic^d^	p-value^e^
**CBCL assessed at 2 years**				
Internalizing score	332	0.54	0.79	0.89
Externalizing score	331	0.63	0.91	0.69
**SRS-2 assessed at 3 years**				
Total score	327	0.72	1.05	0.34
**BRIEF-P assessed at 3 years**				
Inhibition score	325	0.72	1.06	0.34
Shift score	333	0.74	1.08	0.33
Emotional control score	332	0.66	0.97	0.54
Work memory score	324	0.50	0.73	0.94
Plan and organization score	330	0.78	1.15	0.20
**WPPSI-IV assessed at 3 years**				
Verbal comprehension score	310	0.76	1.11	0.27
Visuospatial score	310	0.70	1.02	0.40
Work memory score	310	0.53	0.78	0.90
Total score	310	0.53	0.77	0.89

a Child Behavior Checklist (CBCL), Social Responsiveness Scale (SRS-2), Behavior Rating Inventory of Executive Function, Pre-school (BRIEF-P) and Wechsler Preschool and Primary Scale of Intelligence (WPPSI-IV) were coded as categorical variables (1st tertile, 2nd tertile and 3rd tertile).

b Number of observations.

c Variance in β-diversity that can be explained by differences in neurodevelopmental parameters between the different levels; adjusted for child age at neurodevelopment assessment (except for WPPSI-IV scores that were already standardized on child age and on the neuropsychologist who conducted the test), delivery mode, gestational duration, child characteristics (sex, weight and length at birth), breastfeeding duration, period of introduction of solid food, antibiotics use during the first year of life, presence of pets at home, maternal parity, child perinatal passive smoking up to one year, HOME questionnaire total score, main mode of child care at 12 months and maternal characteristics (parity, age and BMI before pregnancy, education, and smoking status during pregnancy, maternal anxiety and depression score during the third trimester of pregnancy).

d Ratio of the mean square between neurodevelopment level groups due to the neurodevelopment level to the mean square within neurodevelopment level groups (error mean square).

e After correction for multiple testing, the significance threshold was 0.002.

### 3.4 Relation between gut microbiota taxonomy and child neurodevelopment

The number of children included in the taxonomy analysis varied between 315 and 338. After correction for multiple testing, the common significance threshold for phyla and genera analyses was 0.0002.

#### 3.4.1 Phylum level

**Fig. 1:**
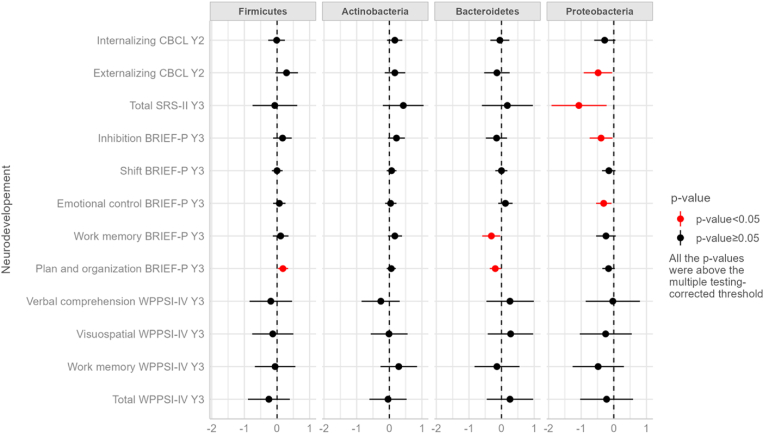
Estimated effects of the abundance of the 4 most abundant phyla in the child gut microbiota at one year of age on neurodevelopment at two and three years in the SEPAGES cohort (sample sizes between 315 and 338). Average changes in neurodevelopment scores when the phylum relative abundance increase by 10 %; adjusted for child age at neurodevelopment assessment (except for WPPSI-IV scores that were already standardized), delivery mode, gestational duration, child characteristics (sex, weight and length at birth), breastfeeding duration, period of introduction of solid food, antibiotics use during the first year of life, presence of pets at home, maternal parity, child perinatal passive smoking up to one year, HOME questionnaire total score, main mode of child care at 12 months and maternal characteristics (parity, age and BMI before pregnancy, education, and smoking status during pregnancy, maternal anxiety and depression score during the third trimester of pregnancy). See Table 4 for point estimates. Child Behavior Checklist (CBCL), Social Responsiveness Scale (SRS-2) and Behavior Rating Inventory of Executive Function, Pre-school (BRIEF-P) are continuous raw scores. Wechsler Preschool and Primary Scale of Intelligence (WPPSI-IV) are continuous scores standardized on child age at evaluation and on the neuropsychologist who conducted the test. After correction for multiple testing, the significance threshold was 0.0002. Y2: scores assessed at 2 years; Y3: scores assessed at 3 years. (For interpretation of the references to colour in this figure legend, the reader is referred to the Web version of this article.)

None of the associations between the 4 most abundant phyla and the 12 neurodevelopment score variables passed the threshold corrected for multiple testing. No association was observed with WPPSI-IV scores. Associations of Proteobacteria with lower scores of different neurodevelopmental parameters, including CBCL externalizing problem, total SRS-2 social behavior problem, BRIEF-P inhibition and emotional control problems did not pass the multiple-testing-corrected significance threshold (p-values: 0.03, 0.01, 0.03 and 0.01, respectively). Two phyla tended to be (non-significantly) associated with higher BRIEF-P scores: a 10 % increase in Firmicutes tended to be associated with more planning and organizational problems (p-value: 0.02), while a 10 % increase in Bacteroidetes tended to be associated with a decrease in planning and organizational and working memory problems (p-values: 0.04 and 0.03, respectively). Cohen's f^2^ were not in favor of gut microbiota phyla explaining a large share in the variability of neurodevelopmental scores (Cohen's f^2^ < 0.1, Table 4). Detailed results are given in Table 4 and Fig. 1.

**Table 4:** Adjusted associations between the abundance of the 4 most abundant phyla in the child gut microbiota at one year of age and the child neurodevelopment at two and three years in the SEPAGES cohort (sample sizes between 315 and 338).

Neurodevelopmental scores^a^ and phyla relative abundances	N^b^	Beta^c^	95 % CI^d^	p-value^e^	Cohen's *f*^f^
**CBCL assessed at 2 years**					
**Internalizing score**	336				
Firmicutes		−0.01	−0.27, 0.24	0.92	<0.001
Actinobacteria		0.17	−0.07, 0.40	0.16	0.006
Bacteroidetes		−0.05	−0.34, 0.24	0.73	<0.001
Proteobacteria		−0.28	−0.60, 0.05	0.10	0.009
**Externalizing score**	335				
Firmicutes		0.29	−0.05, 0.64	0.09	0.009
Actinobacteria		0.17	−0.14, 0.49	0.28	0.004
Bacteroidetes		−0.14	−0.53, 0.25	0.48	0.002
Proteobacteria		−0.48	−0.92, −0.05	0.03	0.02

**SRS-2 assessed at 3 years**	332				
**Total score**					
Firmicutes		−0.07	−0.75, 0.62	0.85	<0.001
Actinobacteria		0.43	−0.20, 1.05	0.18	0.006
Bacteroidetes		0.18	−0.60, 0.96	0.66	<0.001
Proteobacteria		−1.07	−1.91, −0.22	0.01	0.02

**BRIEF-P assessed at 3 years**					
**Inhibition score**	330				
Firmicutes		0.17	−0.12, 0.45	0.25	0.004
Actinobacteria		0.22	−0.04, 0.48	0.10	0.009
Bacteroidetes		−0.15	−0.48, 0.17	0.36	0.003
Proteobacteria		−0.39	−0.74, −0.04	0.03	0.02
**Shift score**	338				
Firmicutes		0.00	−0.16, 0.17	0.95	<0.001
Actinobacteria		0.07	−0.08, 0.22	0.35	0.003
Bacteroidetes		0.00	−0.19, 0.18	0.97	<0.001
Proteobacteria		−0.15	−0.36, 0.05	0.13	0.007
**Emotional control score**	337				
Firmicutes		0.07	−0.12, 0.26	0.50	0.002
Actinobacteria		0.04	−0.13, 0.22	0.63	<0.001
Bacteroidetes		0.12	−0.10, 0.34	0.27	0.004
Proteobacteria		−0.31	−0.54, −0.07	0.01	0.02
**Work memory score**	329				
Firmicutes		0.11	−0.13, 0.35	0.35	0.003
Actinobacteria		0.17	−0.04, 0.39	0.12	0.008
Bacteroidetes		−0.31	−0.59, −0.04	0.03	0.02
Proteobacteria		−0.24	−0.54, 0.06	0.12	0.008
**Plan and organization score**	335				
Firmicutes		0.18	0.03, 0.34	0.02	0.02
Actinobacteria		0.06	−0.08, 0.20	0.40	0.002
Bacteroidetes		−0.19	−0.36, −0.01	0.04	0.01
Proteobacteria		−0.16	−0.35, 0.03	0.11	0.009

**WPPSI-IV assessed at 3 years**					
**Verbal comprehension score**	315				
Firmicutes		−0.19	−0.84, 0.46	0.56	0.001
Actinobacteria		−0.26	−0.85, 0.32	0.38	0.003
Bacteroidetes		0.26	−0.46, 0.99	0.47	0.002
Proteobacteria		−0.03	−0.86, 0.80	0.94	<0.001
**Visuospatial score**	315				
Firmicutes		−0.13	−0.76, 0.50	0.68	<0.001
Actinobacteria		−0.01	−0.57, 0.56	0.99	<0.001
Bacteroidetes		0.28	−0.42, 0.97	0.43	0.002
Proteobacteria		−0.25	−1.04, 0.55	0.55	0.001
**Work memory score**	315				
Firmicutes		−0.06	−0.68, 0.56	0.85	<0.001
Actinobacteria		0.29	−0.27, 0.85	0.31	0.004
Bacteroidetes		−0.14	−0.83, 0.55	0.69	<0.001
Proteobacteria		−0.48	−1.26, 0.31	0.23	0.005
**Total score**	315				
Firmicutes		−0.25	−0.89, 0.39	0.44	0.002
Actinobacteria		−0.04	−0.61, 0.53	0.89	<0.001
Bacteroidetes		0.26	−0.45, 0.97	0.47	0.002
Proteobacteria		−0.22	−1.03, 0.59	0.59	<0.001

a Child Behavior Checklist (CBCL), Social Responsiveness Scale (SRS-2) and Behavior Rating Inventory of Executive Function, Pre-school (BRIEF-P) are continuous raw scores. Wechsler Preschool and Primary Scale of Intelligence (WPPSI-IV) are continuous scores standardized on child age at evaluation and on the neuropsychologist who conducted the test.

b Number of observations.

c Average changes in neurodevelopment scores when the phylum relative abundance increased by 10 %; adjusted for child age at neurodevelopment assessment (except for WPPSI-IV scores that were already standardized), delivery mode, gestational duration, child characteristics (sex, weight and length at birth), breastfeeding duration, period of introduction of solid food, antibiotics use during the first year of life, presence of pets at home, maternal parity, child perinatal passive smoking up to one year, HOME questionnaire total score, main mode of child care at 12 months and maternal characteristics (parity, age and BMI before pregnancy, education, and smoking status during pregnancy, maternal anxiety and depression score during the third trimester of pregnancy).

d CI: Confidence Interval.

e After correction for multiple testing, the significance threshold was 0.0002.

f Cohen's f is calculated as (R^2^_full_ - R^2^_reduced_)/(1-R^2^_full_), where R^2^_full_ is the R^2^ of the model that includes the gut microbiota parameter and the covariates as explanatory variables and R^2^_reduced_ is the R^2^ of the model with only the covariates.

#### 3.4.2 Genus level

**Fig. 2:**
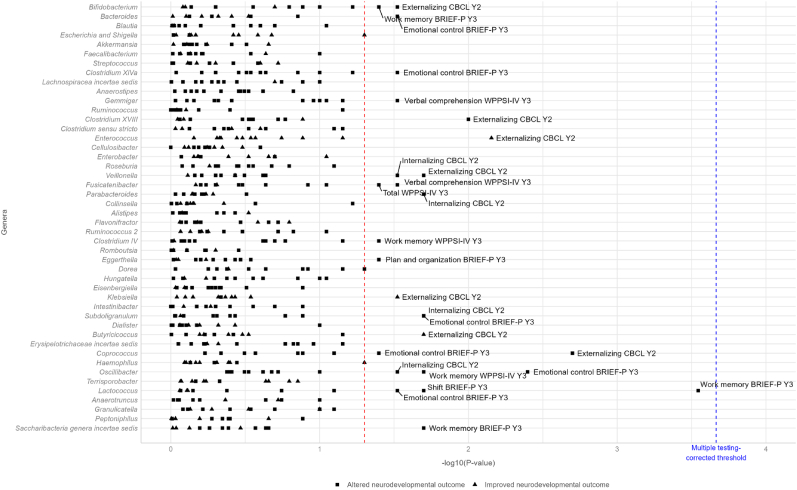
Estimated effects of abundance of the 46 most abundant genera in the child gut microbiota at one year of age on the neurodevelopment at two and three years in the SEPAGES cohort (sample sizes between 315 and 338). -log10(p-value) of the associations between the 46 most abundant genera (detected in at least 30 % of the children) and the neurodevelopment scores when the relative abundance of a genus (%) is multiplied by Euler number e, adjusted for child age at neurodevelopment assessment (except for WPPSI-IV scores that were already standardized), delivery mode, gestational duration, child characteristics (sex, weight and length at birth), breastfeeding duration, period of introduction of solid food, antibiotics use during the first year of life, presence of pets at home, maternal parity, child perinatal passive smoking up to one year, HOME questionnaire total score, main mode of child care at 12 months and maternal characteristics (parity, age and BMI before pregnancy, education, and smoking status during pregnancy, maternal anxiety and depression score during the third trimester of pregnancy). Child Behavior Checklist (CBCL), Social Responsiveness Scale (SRS-2) and Behavior Rating Inventory of Executive Function, Pre-school (BRIEF-P) are continuous raw scores. Wechsler Preschool and Primary Scale of Intelligence (WPPSI-IV) are continuous scores standardized on child age at evaluation and on the neuropsychologist who conducted the test. The vertical dotted red line represents a p-value equal to 0.05. The vertical dotted blue line represents the multiple testing corrected p-value: 0.0002. Y2: scores assessed at 2 years; Y3: scores assessed at 3 years. Complete results of the associations between the 46 most abundant genera and the neurodevelopmental parameters are given in Table S4. (For interpretation of the references to colour in this figure legend, the reader is referred to the Web version of this article.)

**Fig. 3:**
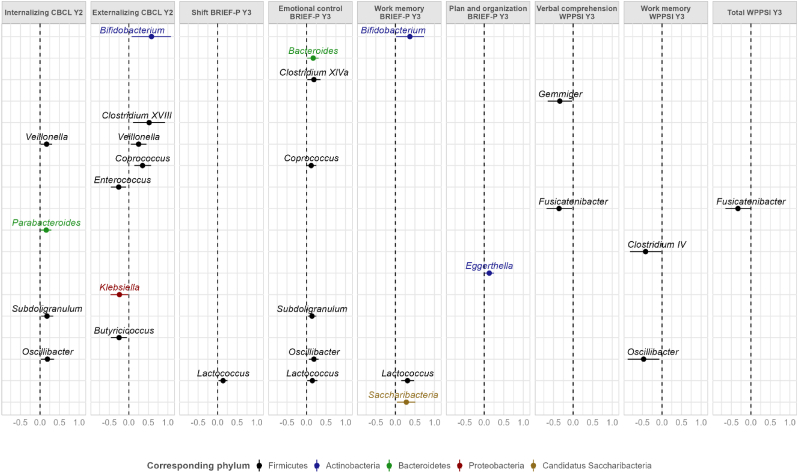
Strongest estimated effects (p < 0.05) of increased abundance of the 46 most abundant genera of the child gut microbiota at one year of age on the neurodevelopment at two and three years in the SEPAGES cohort (sample sizes between 315 and 338). Average changes in neurodevelopment scores when the relative abundance of a genus (%) is multiplied by Euler number e, adjusted for child age at neurodevelopment assessment (except for WPPSI-IV scores that were already standardized), delivery mode, gestational duration, child characteristics (sex, weight and length at birth), breastfeeding duration, period of introduction of solid food, antibiotics use during the first year of life, presence of pets at home, maternal parity, child perinatal passive smoking up to one year, HOME questionnaire total score, main mode of child care at 12 months and maternal characteristics (parity, age and BMI before pregnancy, education, and smoking status during pregnancy, maternal anxiety and depression score during the third trimester of pregnancy). Child Behavior Checklist (CBCL), Social Responsiveness Scale (SRS-2) and Behavior Rating Inventory of Executive Function, Pre-school (BRIEF-P) are continuous raw scores. Wechsler Preschool and Primary Scale of Intelligence (WPPSI-IV) are continuous scores standardized on child age at evaluation and on the neuropsychologist who conducted the test. After correction for multiple testing, the significance threshold was 0.0002. Complete results of the associations between the 46 most abundant genera and the neurodevelopmental parameters are given in Table S4. (For interpretation of the references to colour in this figure legend, the reader is referred to the Web version of this article.)

None of the associations between the 46 most abundant genera and the 12 neurodevelopment score variables passed the threshold corrected for multiple testing (p-value<0.0002). Greater abundances of genera *Clostridium XVIII*, *Veillonella* and *Coprococcus* tended to be associated with higher CBCL externalizing problem score (p-values: 0.01, 0.02 and 0.002, respectively) and greater abundances of genera *Parabacteroides* and *Subdoligranulum* tended to be associated with higher CBCL internalizing problem score (p-values: 0.02 and 0.02, respectively), while a greater abundance of genera *Enterococcus* and *Butyricicoccus* tended to be associated lower CBCL externalizing problem (p-values: 0.007 and 0.02, respectively). A greater abundance of genera *Subdoligranulum* and *Oscillibacter* tended to be associated with higher BRIEF-P score of emotional control problem (p-values: 0.02 and 0.004, respectively) while a greater abundance of *Lactococcus* genus tended to be associated with higher BRIEF-P shift problem score (p-value: 0.02). Finally, greater abundances of genera *Lactococcus* and *Saccharibacteria genera incertae sedis* tended to be associated with increased BRIEF-P working memory problem score (p-values: 0.0003 and 0.02, respectively) while greater abundance of genus *Oscillibacter* tended to be associated with higher WPPSI-IV working memory problem score (p-value: 0.02). The p-values and the direction of the associations according to the neurodevelopment score and bacterial genus are shown Fig. 2. Beta values with 95 % confidence intervals for the strongest associations (p-value<0.05) are given in Fig. 3. Complete results of the genus analyses are shown in Table S4. Cohen's f^2^ were not in favor of gut microbiota genera explaining a large share of the variability in neurodevelopmental scores (Cohen's f^2^ < 0.1, Table S4).

Table 5 shows for each neurodevelopmental parameter a multiple linear regression including all gut microbiota parameters that were associated with this specific outcome in the previous regression models, adjusting for the same covariates. The Cohen's f^2^ values of these models show that the effect sizes of the gut microbiota parameters on the neurodevelopmental scores are mostly small (Cohen's f^2^ < 0.2), but for the externalizing CBCL score (Cohen's f^2^: 0.22, corresponding to a medium effect size).

**Table 5:** Adjusted associations between the gut microbiota parameters at one year of age and the child neurodevelopment at two and three years in the SEPAGES cohort (one multiple linear regression model per outcome, sample sizes between 315 and 338).

Neurodevelopmental scores1 and gut microbiota parameters^a^	N^b^	R^b^ full model^c^	R^b^ reduced model^d^	Cohen's f^e^
**CBCL assessed at 2 years**				
Internalizing score	336	0.24	0.13	0.14
Externalizing score	335	0.26	0.10	0.22
**SRS-2 assessed at 3 years**				
Total score	332	0.22	0.17	0.07
**BRIEF-P assessed at 3 years**				
Inhibition score	330	0.13	0.09	0.05
Shift score	338	0.14	0.09	0.06
Emotional control score	337	0.19	0.09	0.12
Work memory score	329	0.21	0.11	0.13
Plan and organization score	335	0.16	0.10	0.08
**WPPSI-IV assessed at 3 years**				
Verbal comprehension score	315	0.19	0.13	0.08
Visuospatial score	315	0.11	0.06	0.06
Work memory score	315	0.17	0.10	0.08
Total score	315	0.16	0.09	0.08

a Child Behavior Checklist (CBCL), Social Responsiveness Scale (SRS-2) and Behavior Rating Inventory of Executive Function, Pre-school (BRIEF-P) are continuous raw scores. Wechsler Preschool and Primary Scale of Intelligence (WPPSI-IV) are continuous scores standardized on child age at evaluation and on the neuropsychologist who conducted the test.

b Number of observations.

c Proportion of the variation in the neurodevelopmental score that is predictable by the explanatory variables which are phyla Firmicutes, Bacteroidetes and Proteobacteria; and genera *Bifidobacterium, Bacteroides, Clostridium XlVa, Gemmiger, Clostridium XVIII, Enterococcus, Veillonella, Fusicatenibacter, Parabacteroides, Clostridium IV, Eggerthella, Klebsiella, Subdoligranulum, Butyricicoccus, Coprococcus, Oscillibacter, Lactococcus, Saccharibacteria genera incertae sedis*, and the covariates.

d Proportion of the variation in the neurodevelopmental score that is predictable by the covariates.

e Cohen's f is calculated as (R^2^full - R^2^reduced)/(1-R^2^full), where R^2^full is the coefficient of determination of the model that includes the gut microbiota parameters and the covariates as explanatory variables and R^2^reduced is the coefficient of determination of the model with only the covariates.

### 3.5 Sensitivity analyses

Using another threshold (10,000 instead of 5,000) of rarefaction to define α-diversity indices did not strongly influence our results (Table S5). Not adjusting on the HOME variable (assessed at 3 years) in the multiple linear regression models on the CBCL scores (assessed at 2 years) did not strongly change our results either (Table S6). Likewise, standardizing or not the WPPSI-IV variables for the neuropsychologist who conducted the test did not change the results. For the sake of consistency with previous studies on SEPAGES cohort data, we presented the results obtained with WPPSI-IV variables standardized for the neuropsychologist in the main analyses (Table S7). Not standardizing the gut microbiota variables for technical factors (child age at stool collection and MiSeq sequencing batch effect when associated with gut microbiota parameters with a p-value<0.2) did not change the results. Not adjusting the statistical models on gut microbiota predictors that are not *a priori* predictors of neurodevelopment (delivery mode, antibiotics use during the first year of life and presence of pets at home) did not change the results either (Table S8). Our analyses suggested non-monotonic relations for specific richness with visuospatial WPPSI-IV score, for Shannon diversity with plan and organization BRIEF-P score, for Bacteroidetes phylum for inhibition BRIEF-P score, and for some genera (*Bacteroides*, *Romboutsia* and *Eggerthella*) with different behavior and executive function scores (CBCL, SRS-II and BRIEF-P tests, Table S9).

## 4 Discussion

In a study of 356 families, there was no clear evidence of association between the gut microbiota assessed at one year and neurodevelopmental scores at the ages of 2 and 3. Some weak trends (not passing the multiple testing corrected significance threshold) are worth reporting as exploratory results: without correcting for multiple testing, an increase in Firmicutes was associated with more planning and organization problems at age 3, as measured by the BRIEF-P test. An increase in Bacteroidetes and Proteobacteria was associated with greater socio-emotional neurodevelopment, assessed by different tests. Specific genera such as *Lactococcus*, *Coprococcus, Oscillibacter, Clostridium XVIII*, *Veillonella, Parabacteroides, Subdoligranulum* and *Saccharibacteria genera incertae sedis* tended to be associated with poorer socio-emotional development, while genera such as *Enterococcus* and *Butyricicoccus* tended to be associated with greater socio-emotional development, assessed by different scores. None of these associations were maintained after correction for multiple testing. The explanatory power of the considered gut microbiota parameters on the variability of neurodevelopmental scores at 2 or 3 years (quantified by the models’ R^2^) was low, which was not in favor of variations in the gut microbiota assessed at one year explaining a large share of neurodevelopmental scores.

While our primary interpretation was based on results corrected for multiple testing, we also acknowledge that some associations, though not reaching the corrected threshold for multiple testing, may suggest potential patterns of interest. In an exploratory manner, we will briefly discuss these uncorrected results to provide insights that could inform future research. Similarly to our study, Laue et al. found no significant link between the Shannon α-diversity index or β-diversity (measured at 6 weeks, 1, 2, and 3 years) and the total SRS-2 score of social behavior problem at 3 years in 150 children; an exception was noted for the β-diversity at 2 years, which was correlated with the total SRS-2 scores at 3 years (Laue et al., 2020). Sordillo et al. did not find a significant association between Shannon α-diversity index assessed at 3 and 6 months and child general development assessed with the ASQ-3 at age 3 in 309 children (Sordillo et al., 2019). Aatsinki et al. did not find an association between α-diversity (Shannon and Chao indices assessed at 2.5 months) and infant temperament assessed by the IBQ-S questionnaire in 301 infants aged 6 months (Aatsinki et al., 2019); Carlson et al. also did not find an association between α-diversity (Chao1, observed species, Shannon, and Faith indices) assessed at one year and the child cognitive development assessed by the Mullen scale at the same age, but found a negative correlation between Chao1, observed species and Faith phylogenetic α-diversity indices and early learning cognitive composite score, in 89 children (Carlson et al., 2018). Loughman et al. found no evidence of an association between 1- and 6-month infant gut microbiota α-diversity (assessed Shannon, Simpson, Chao1, and observed species) and child behaviors at 2 years assessed by the CBCL test, nor with the β-diversity (assessed by weighted and unweighted UniFrac distance); they found a weak association between higher α-diversity (all studied indices) at 12 months with increased risk of behavior problems at 2 years in 201 children (Loughman et al., 2020).

All the studies mentioned, including ours, are prospective epidemiological studies in children from the general population. These studies suggest that the variations in gut microbiota α- and β-diversity among neurotypical children may be too small to induce large changes in neurodevelopmental scores. Case-control studies of gut microbiota diversity in ASD or ADHD children compared to neurotypical children reported either no associations (Dan et al., 2020; Ding et al., 2021; Ha et al., 2021; Jiang et al., 2018; Kushak et al., 2017; Pulikkan et al., 2018; Richarte et al., 2021; Szopinska-Tokov et al., 2020; Wang et al., 2020, 2022; Zhang et al., 2018), positive associations (Chen et al., 2020; Ding et al., 2020; Li et al., 2019; Wang et al., 2020; Zhai et al., 2019; Zou et al., 2020; Zurita et al., 2020) or negative associations (Kang et al., 2018; Liu et al., 2019; Ma et al., 2019; Prehn-Kristensen et al., 2018). These retrospective studies should be interpreted with caution due to the risk of reverse causality. Indeed, some studies suggest that it is the dietary preferences of ASD patients that lead to differences in gut microbiota diversity and composition with neurotypical patients (Yap et al., 2021).

In our study, none of the associations between taxonomy and neurodevelopment passed the threshold corrected for multiple testing (p < 0.0002). Without correction for multiple testing, Bacteroidetes phylum was associated with less working memory and plan and organization problems (BRIEF-P scores, p-values: 0.03 and 0.04, respectively), which was in line with the results of some previous prospective epidemiological studies (Carlson et al., 2018; Loughman et al., 2020; Tamana et al., 2021). No previous epidemiological studies have shown a positive association between the relative abundance of Proteobacteria and neurodevelopment in children, so that replication is warranted.

Higher relative abundance of *Coprococcus* at 2 years was associated with poorer social behaviors assessed with the SRS-2 total score at 3 years in the study of Laue et al. (2020), and while we did not find an association between *Coprococus* and SRS-2, we did find *Coprococcus* at 1 year associated with higher CBCL externalizing score at 2 years. Guzzardi et al. found that higher abundance of genus *Veillonella* in the first-pass meconium sample was an important determinant of higher practical reasoning scores at 5 years (Guzzardi et al., 2022) and Laue et al. found that higher relative abundance of *Butyricicoccus pullicaecorum* at 3 years was associated with worse social behavior at 3 years (Laue et al., 2020), contrary to our results that suggested an association between the abundance of genus *Veillonella* and poorer neurodevelopment and an association between genus *Butyricicoccus* and greater neurodevelopment. In case-control studies, *Veillonella* relative abundance was higher in children with ASD (Strati et al., 2017; Zhang et al., 2018) and ADHD (Wan et al., 2020), compared to neurotypical children. To our best knowledge, no associations were found in previous prospective epidemiological studies between the relative abundance of the genera *Lactococcus*, *Oscillibacter*, *Enterococcus*, *Clostridium XVIII*, *Parabacteroides* or *Subdoligranulum* and neurodevelopment parameters. Variations in study design, including sample size, participant age, selection of confounding factors and sequencing techniques (e.g., choice of primers and taxonomic classification methods), may contribute to discrepancies between studies. Addressing these challenges requires standardized methodological guidelines for epidemiological studies on the gut microbiota–brain axis to enhance consistency and reproducibility across research efforts.

Several studies causally implicating the gut microbiota in the regulation of neurodevelopment have been described in rodent models, as reviewed by Morais et al. (2021). The challenges in understanding the underlying mechanisms in this field are related to the limitations of animal models attempting to replicate human diseases, and the lack of prospective epidemiological studies with sufficient sample sizes.

Certain bacterial genera, which could be considered biologically beneficial for neurodevelopment, tended to be associated with impaired neurodevelopment in our study. For example, *Lactococcus lactis* (Nardi et al., 2002)*,* certain *Coprococcus* species (King et al., 2019) and certain *Oscillibacter* species (Iino et al., 2007; Lee et al., 2013) have been described as producers of short-chain fatty acids. Numerous *in vitro* studies have demonstrated beneficial effect of short-chain fatty acids on the central nervous system (Kassan et al., 2023; Yang et al., 2019). The possible mechanisms of action are complex and probably take place from the intestinal mucosa by activating intermediate signals (Cryan et al., 2019; Lecerf and Delzenne, 2021). Although these bacteria have been described as beneficial in some studies, it is worth noting that there may be a non-linear relationship between the abundance of the bacterial taxa and neurodevelopment. For example, high concentrations of short-chain fatty acid propionic acid were reported as neurotoxic and implicated in autism-like phenotypes in animal models (El-Ansary et al., 2012; MacFabe et al., 2007). Indeed, our sensitivity analyses, which must be considered in the light of the large number of tests done, suggest that certain parameters of the gut microbiota might have a non-monotonic relationship with neurodevelopment (Table S9).

Our study is based on a prospective couple child cohort including 360 families with child gut microbiota and neurodevelopment assessment, and rich questionnaire data to assess potential confounders. DNA extraction from fecal samples was performed using a method validated to accurately reflect the overall microbial community, as confirmed by the Metagenomics of the Human Intestinal Tract project and International Human Microbiome Standards (Cardona et al., 2012; Metagenomics of the Human Intestinal Tract (METAHIT Project); Santiago et al., 2014). Assessing different neurodevelopmental characteristics, and at different periods of development, enabled us to assess both socio-emotional and cognitive domains of neurodevelopment. Although we adjusted for confounders in our analyses, we cannot completely rule out the possibility of residual confounding. Notably, a more precise adjustment of the associations for diet than what the SEPAGES cohort data allowed could have strengthened the models, as children's dietary patterns are likely a significant source of bias (Johnson and Howell, 2021). Women in the SEPAGES cohort tended to be more educated, were older, and smoked less compared to the average pregnant woman in France (Lyon-Caen et al., 2019). This does not undermine the relations suggested in our study but could have limited the ability to study interactions with socially-related factors (which may anyway require much larger populations), which was not our objective. Additionally, the homogeneity of our population helped to reduce the risk of confounding biases. In spite of the relatively large sample size, the power of our study to detect weak associations was consequently probably low. Larger cohort studies or longitudinal designs could help clarify the potentially weak link between gut microbiota composition and neurodevelopment by providing a larger sample size and, consequently, greater statistical power to strengthen the confidence in our findings. The lack of statistical significance in our study could also be explained by limited variability in neurodevelopmental scores, as our study focused on mostly neurotypical children, compared to what is observed in case-controls studies involving patients. In contrast, much of the epidemiological evidence supporting a role for gut microbiota in neurodevelopment comes from cross-sectional case-control studies. Future prospective case-control studies examining the role of the gut microbiota on neurodevelopmental disorders, such as ASD or ADHD, could provide further insights. Lastly, while 16S rRNA gene sequencing provided valuable insights, future studies using whole genome sequencing could assess functional changes and species-level impacts, and integrating metabolomic approaches could help identify key microbial metabolites involved in neurodevelopmental processes, which may be crucial for understanding the microbiota-gut-brain axis in human.

In our couple-child cohort, suggestive associations were observed between child gut microbiota taxonomy and neurodevelopment, but not maintained after correction for multiple comparison. Our study does not favor the gut microbiota, as assessed at one year of age, exerting a strong influence on neurodevelopmental scores at 2 and 3 years of age. Larger studies are needed to examine a possibly weak link between the gut microbiota of one-year children from the general population on their neurodevelopment.

## CRediT authorship contribution statement

**Aline Davias:** Writing – original draft, Formal analysis, Methodology. **Sarah Lyon-Caen:** Data curation, Writing – review & editing, Supervision, Conceptualization, Validation, Project administration. **Nina Iszatt:** Writing – review & editing, Methodology. **Celine Monot:** Methodology, Writing – review & editing. **Yamina Rayah:** Methodology, Writing – review & editing. **Zehra Esra Ilhan:** Writing – review & editing, Methodology. **Karine Guichardet:** Writing – review & editing, Methodology. **Sam Bayat:** Writing – review & editing. **Séverine Valmary-Degano:** Methodology, Writing – review & editing. **Gina Muckle:** Methodology, Writing – review & editing. **Merete Eggesbø:** Writing – review & editing, Conceptualization, Investigation, Project administration, Funding acquisition. **Patricia Lepage:** Investigation, Writing – review & editing, Methodology, Funding acquisition, Project administration, Conceptualization. **Claire Philippat:** Project administration, Funding acquisition, Conceptualization, Validation, Methodology, Writing – review & editing, Supervision, Investigation, Formal analysis. **Rémy Slama:** Writing – original draft, Project administration, Conceptualization, Validation, Methodology, Funding acquisition, Writing – review & editing, Supervision, Investigation, Formal analysis.

## Declaration of interest statement

The authors declare that they have no known competing financial interests or personal relationships that could have appeared to influence the work reported in this paper. BRIEF-P test was used with permission of the publisher Hogrefe France.

## Data availability statement

The authors do not have permission to share data.

## Fundings

This work was funded by the French Research Agency - ANR (GUMME project, ANR-17-CE34-0013). The SEPAGES cohort was supported by the **European Research Council** (N°311765-E-DOHaD), the **European Community's Seventh Framework Program** (FP7/2007-206 - N°308333-892 HELIX), the **European Union's Horizon 2020** research and innovation program (N° 874583 ATHLETE Project, N°825712 OBERON Project), the **French Research Agency - ANR** (PAPER project ANR-12-PDOC-0029-01, SHALCOH project ANR-14-CE21-0007, ANR-15-IDEX-02 and ANR-15-IDEX5, GUMME project ANR-17-CE34-0013, ETAPE project ANR-18-CE36-0005, EDeN project ANR-19-CE36-0003-01, MEMORI project ANR 21-CE34-0022, ORANDANI project ANR-22-CE36-0018), the F**rench Agency for Food, Environmental and Occupational** 10.13039/100018696**Health** **& Safety - ANSES** (10.13039/100017124CNAP project EST-2016-121, PENDORE project EST-2018-1-264, HyPAxE project EST-2019/1/039, PENDALIRE project EST-2022-169), the **Plan Cancer** (Canc’Air project), the **French** 10.13039/100002002**Cancer Research Foundation** **Association de Recherche sur le Cancer –** **ARC****,** the **French Endowment** Fund **AGIR for chronic diseases – APMC** (projects PRENAPAR, LCI-FOT, DysCard), the **French Endowment** Fund **for Respiratory Health**, the F**rench** Fund **– Fondation de France** (CLIMATHES – 00081169, SEPAGES 5–00099903, ELEMENTUM - 00124527**)**. Nina Iszatt is supported by the 10.13039/501100005416Norwegian Research Council grant agreement "NON-PROTECTED” No. 275903/F20.

## Declaration of competing interest

The authors declare that they have no known competing financial interests or personal relationships that could have appeared to influence the work reported in this paper.

## Data Availability

The authors do not have permission to share data.

## Abbreviations

ADHD: Attention-Deficit/Hyperactivity Disorder
ADI-R: the Autism Diagnostic Interview-Revised
ASD: Autism Spectrum Disorders
ASV: Amplicons Sequencing Variants
ASQ: Ages and Stages Questionnaire
BSID-III: Bayley Scales of Infant and Toddler Development, Third edition
BMI: Body Mass Index
BRIEF-P: Behavior Rating Inventory of Executive Function, Pre-school
CBCL: Child Behavior Checklist
CNS: Central Nervous System
DAG: Directed Acyclic Graph
DNA: DeoxyriboNucleic Acid
ENS: Enteric Nervous System
FWER: Family-Wise Error Rate
HOME: Home Observation Measurement of the Environment
IBQ-R: Infant Behavior Questionnaire-Revised
IQ: Intelligence Quotient
IQR: InterQuartile Range
PERMANOVA: Permutational Multivariate Analysis of Variance
rRNA: ribosomal RiboNucleic Acid
SEPAGES: Suivi de l’Exposition à la Pollution Atmosphérique durant la Grossesse et Effet sur la Santé
SRS-2: Social Responsiveness Scale
WPPSI-IV: Wechsler Preschool and Primary Scale of Intelligence, fourth edition
