# Supplementary material for: Associations between the gut microbiota at one-year and neurodevelopment in children from the SEPAGES cohort
Source: Brain Behav Immun Health. 2025 Jul 18;48:101063. doi: 10.1016/j.bbih.2025.101063 (PMC12336685; doi:10.1016/j.bbih.2025.101063)
Supplement: Multimedia component 2 [file mmc2.docx]

**Associations between the gut microbiota at one-year and neurodevelopment in children from the SEPAGES cohort**

**Supplementary files**

Aline Davias, Sarah Lyon-Caen, Nina Iszatt, Celine Monot, Yamina Rayah, Zehra Esra Ilhan, Karine Guichardet, Sam Bayat, Séverine Valmary-Degano, Gina Muckle, Merete Eggesbø, Patricia Lepage, Claire Philippat, Rémy Slama

[Table S1: Distribution of α-diversity and major taxa in one year child gut microbiota from SEPAGES cohort. 3](#_Toc184288766)

[Table S2: Distribution of the child neurodevelopment scores in children from SEPAGES cohort. 5](#_Toc184288767)

[Table S3: Estimated effects of maternal and child characteristics on the neurodevelopment at two and three years in the SEPAGES cohort (sample sizes between 315 and 338). 6](#_Toc184288768)

[Table S4: Estimated effects of increased abundance of the 46 most abundant genera of the child gut microbiota at one year of age on the neurodevelopment at two and three years in the SEPAGES cohort (sample sizes between 315 and 338). 6](#_Toc184288769)

[Table S5: Sensitivity analysis – Estimated effects of increased α-diversity indices at different sequencing depths on the neurodevelopment at two and three years in the SEPAGES cohort. 7](#_Toc184288770)

[Table S6: Sensitivity analysis – Effects of including the HOME covariate (assessed at 3 years) in the models of the estimated effects of the gut microbiota parameters on the CBCL scores assessed at 2 years in the SEPAGES cohort. 9](#_Toc184288771)

[Table S7: Sensitivity analysis – Effect of standardization of the WPPSI-IV scores on the neuropsychologist who conducted the test in the models of the estimated effects of the gut microbiota parameters on the WPPSI-IV scores assessed at 3 years in the SEPAGES cohort. 11](#_Toc184288772)

[Table S8: Sensitivity analysis – Effect of standardization of the gut microbiota parameters on the technical factors (child age at feces collection and MiSeq batch effect) in the models of the estimated effects of the gut microbiota parameters on the neurodevelopmental parameters assessed at two and three years in the SEPAGES cohort. 11](#_Toc184288773)

[Table S9: Sensitivity analysis – Investigation of non-monotonic associations between gut microbiota parameters and neurodevelopmental parameters assessed at two and three years in the SEPAGES cohort. 11](#_Toc184288774)

[Figure S1: Directed acyclic graph of the relation between one year child gut microbiota and neurodevelopment at two and three years in the SEPAGES cohort. 12](#_Toc184288775)

[Figure S2: Pearson’s correlations between neurodevelopmental parameters (sample sizes between 303 and 335). 13](#_Toc184288776)

Table S1: Distribution of α-diversity and major taxa in one year child gut microbiota from SEPAGES cohort.

| **Gut microbiota parameters** | **Min.** | **1^st^ quartile** | **Median** | **Mean** | **3^rd^ quartile** | **Max.** | **N** |
| --- | --- | --- | --- | --- | --- | --- | --- |
| **α-diversity indices^1^** |  |  |  |  |  |  |  |
| Specific richness | 18 | 64 | 81 | 83 | 99 | 184 | 350 |
| Shannon diversity | 0.38 | 2.25 | 2.60 | 2.55 | 2.93 | 3.77 | 350 |
| **Phylum^2^** |  |  |  |  |  |  |  |
| *Genus*^2^ |  |  |  |  |  |  |  |
| **Firmicutes** | 0.86 | 22.28 | 35.61 | 38.11 | 51.39 | 90.71 | 356 |
| *Blautia* | 0.00 | 1.34 | 4.12 | 8.16 | 11.20 | 67.83 | 356 |
| *Lachnospiracea incertae sedis* | 0.00 | 0.43 | 1.22 | 2.28 | 2.99 | 30.47 | 356 |
| *Faecalibacterium* | 0.00 | 0.01 | 0.99 | 3.46 | 4.18 | 59.82 | 356 |
| *Clostridium XlVa* | 0.00 | 0.28 | 0.91 | 2.38 | 2.54 | 33.24 | 356 |
| *Clostridium XVIII* | 0.00 | 0.28 | 0.70 | 1.60 | 1.77 | 43.79 | 356 |
| *Streptococcus* | 0.00 | 0.13 | 0.65 | 2.56 | 2.55 | 42.40 | 356 |
| *Anaerostipes* | 0.00 | 0.04 | 0.51 | 2.23 | 2.50 | 34.03 | 356 |
| *Flavonifractor* | 0.00 | 0.05 | 0.22 | 0.44 | 0.59 | 4.86 | 356 |
| *Cellulosibacter* | 0.00 | 0.00 | 0.19 | 1.12 | 1.36 | 24.26 | 356 |
| *Clostridium sensu stricto* | 0.00 | 0.02 | 0.16 | 1.51 | 0.92 | 54.27 | 356 |
| *Intestinibacter* | 0.00 | 0.01 | 0.09 | 0.22 | 0.25 | 3.21 | 356 |
| *Clostridium IV* | 0.00 | 0.01 | 0.07 | 0.39 | 0.28 | 8.44 | 356 |
| *Veillonella* | 0.00 | 0.00 | 0.06 | 0.89 | 0.51 | 39.43 | 356 |
| *Enterococcus* | 0.00 | 0.00 | 0.04 | 1.15 | 0.38 | 28.32 | 356 |
| *Erysipelotrichaceae incertae sedis* | 0.00 | 0.00 | 0.04 | 0.15 | 0.14 | 4.49 | 356 |
| *Romboutsia* | 0.00 | 0.00 | 0.04 | 0.37 | 0.25 | 24.95 | 356 |
| *Ruminococcus 2* | 0.00 | 0.00 | 0.03 | 0.41 | 0.33 | 8.99 | 356 |
| *Roseburia* | 0.00 | 0.00 | 0.03 | 0.96 | 0.58 | 29.17 | 356 |
| *Ruminococcus* | 0.00 | 0.00 | 0.01 | 1.61 | 1.80 | 36.90 | 356 |
| *Butyricicoccus* | 0.00 | 0.00 | 0.01 | 0.17 | 0.14 | 4.03 | 356 |
| *Terrisporobacter* | 0.00 | 0.00 | 0.01 | 0.12 | 0.06 | 6.62 | 356 |
| *Coprococcus* | 0.00 | 0.00 | 0.00 | 0.15 | 0.08 | 12.37 | 356 |
| *Gemmiger* | 0.00 | 0.00 | 0.00 | 1.89 | 0.05 | 48.67 | 356 |
| *Fusicatenibacter* | 0.00 | 0.00 | 0.00 | 0.86 | 0.46 | 24.38 | 356 |
| *Dorea* | 0.00 | 0.00 | 0.00 | 0.32 | 0.31 | 5.65 | 356 |
| *Hungatella* | 0.00 | 0.00 | 0.00 | 0.30 | 0.10 | 19.55 | 356 |
| *Eisenbergiella* | 0.00 | 0.00 | 0.00 | 0.24 | 0.03 | 8.01 | 356 |
| *Subdoligranulum* | 0.00 | 0.00 | 0.00 | 0.21 | 0.13 | 14.73 | 356 |
| *Dialister* | 0.00 | 0.00 | 0.00 | 0.20 | 0.01 | 21.12 | 356 |
| *Oscillibacter* | 0.00 | 0.00 | 0.00 | 0.12 | 0.04 | 11.10 | 356 |
| *Lactococcus* | 0.00 | 0.00 | 0.00 | 0.11 | 0.01 | 7.55 | 356 |
| *Anaerotruncus* | 0.00 | 0.00 | 0.00 | 0.03 | 0.01 | 1.59 | 356 |
| *Granulicatella* | 0.00 | 0.00 | 0.00 | 0.01 | 0.01 | 0.68 | 356 |
| *Peptoniphilus* | 0.00 | 0.00 | 0.00 | 0.01 | 0.00 | 0.86 | 356 |
| **Actinobacteria** | 0.02 | 10.82 | 26.20 | 30.01 | 47.79 | 97.63 | 356 |
| *Bifidobacterium* | 0.02 | 9.60 | 24.93 | 28.97 | 45.84 | 97.63 | 356 |
| *Eggerthella* | 0.00 | 0.05 | 0.19 | 0.35 | 0.42 | 3.56 | 356 |
| *Collinsella* | 0.00 | 0.00 | 0.00 | 0.59 | 0.45 | 23.65 | 356 |
| **Bacteroidetes** | 0.00 | 2.72 | 10.43 | 17.41 | 28.35 | 80.30 | 356 |
| *Bacteroides* | 0.00 | 1.84 | 9.09 | 15.57 | 25.54 | 77.75 | 356 |
| *Parabacteroides* | 0.00 | 0.00 | 0.02 | 0.70 | 0.45 | 39.72 | 356 |
| *Alistipes* | 0.00 | 0.00 | 0.00 | 0.49 | 0.20 | 23.98 | 356 |
| **Proteobacteria** | 0.00 | 0.40 | 1.63 | 8.34 | 6.84 | 93.32 | 356 |
| *Escherichia* and *Shigella* | 0.00 | 0.16 | 0.94 | 6.59 | 5.39 | 93.26 | 356 |
| *Enterobacter* | 0.00 | 0.00 | 0.00 | 1.10 | 0.06 | 70.96 | 356 |
| *Klebsiella* | 0.00 | 0.00 | 0.00 | 0.23 | 0.03 | 15.55 | 356 |
| *Haemophilus* | 0.00 | 0.00 | 0.00 | 0.14 | 0.02 | 15.48 | 356 |
| **Verrucomicrobia** |  |  |  |  |  |  |  |
| *Akkermansia* | 0.00 | 0.00 | 0.03 | 6.06 | 3.43 | 82.62 | 356 |
| **Candidatus Saccharibacteria** |  |  |  |  |  |  |  |
| *Saccharibacteria genera incertae sedis* | 0.00 | 0.00 | 0.00 | 0.01 | 0.01 | 0.26 | 356 |
| ^1^Calculated on the ASV rarefied data with a threshold of 5,000 reads (n=350).  ^2^Distribution of phyla and their most abundant genera, calculated on the relative abundance ASV data (n=356). | | | | | | | |

Table S2: Distribution of the child neurodevelopment scores in children from SEPAGES cohort.

| **Neurodevelopmental scores^1^** | **Min.** | **1^st^ quartile** | **Median** | **Mean** | **3^rd^ quartile** | **Max.** | **N** |
| --- | --- | --- | --- | --- | --- | --- | --- |
| **CBCL assessed at 2 years** |  |  |  |  |  |  |  |
| Internalizing score | 0 | 3 | 6 | 6.8 | 9 | 29 | 336 |
| Externalizing score | 1 | 9 | 12 | 13.1 | 17 | 34 | 335 |
| **SRS-2 assessed at 3 years** |  |  |  |  |  |  |  |
| Total score | 4 | 20 | 27 | 28.7 | 36 | 80 | 332 |
| **BRIEF-P assessed at 3 years** |  |  |  |  |  |  |  |
| Inhibition score | 16 | 20 | 24 | 23.7 | 27 | 40 | 330 |
| Shift score | 10 | 11 | 12 | 13.3 | 15 | 26 | 338 |
| Emotional control score | 10 | 12 | 14 | 14.7 | 17 | 28 | 337 |
| Work memory score | 17 | 18 | 21 | 21.6 | 24 | 41 | 329 |
| Plan and organization score | 10 | 12 | 14 | 14.0 | 16 | 22 | 335 |
| **WPPSI-IV assessed at 3 years** |  |  |  |  |  |  |  |
| Verbal comprehension score | 81 | 104 | 111 | 112.0 | 119 | 144 | 315 |
| Visuospatial score | 85 | 103 | 109 | 110.2 | 118 | 138 | 315 |
| Work memory score | 75 | 100 | 106 | 106.1 | 113 | 148 | 315 |
| Total score | 84 | 104 | 112 | 112.8 | 120 | 145 | 315 |
| ^1^Child Behavior Checklist (CBCL), Social Responsiveness Scale (SRS-2) and Behavior Rating Inventory of Executive Function, Pre-school (BRIEF-P) are continuous raw scores. Wechsler Preschool and Primary Scale of Intelligence (WPPSI-IV) are continuous scores standardized on child age at evaluation and on the neuropsychologist who conducted the test. | | | | | | | |

Table S3: Estimated effects of maternal and child characteristics on the neurodevelopment at two and three years in the SEPAGES cohort (sample sizes between 315 and 338).

Please refer to the excel file.

Table S4: Estimated effects of increased abundance of the 46 most abundant genera of the child gut microbiota at one year of age on the neurodevelopment at two and three years in the SEPAGES cohort (sample sizes between 315 and 338).

Please refer to the excel file.

Table S5: Sensitivity analysis – Estimated effects of increased α-diversity indices at different sequencing depths on the neurodevelopment at two and three years in the SEPAGES cohort.

| **Neurodevelopmental scores^1^ and α-diversity indices** | **Threshold 5,000 (n=350)^2^** | | | |  | **Threshold 5,000 (n=339)** | | | |  | **Threshold 10,000 (n=339)** | | | |
| --- | --- | --- | --- | --- | --- | --- | --- | --- | --- | --- | --- | --- | --- | --- |
|  | **N^3^** | **Beta^4^** | **95% CI^5^** | **p-value^6^** |  | **N^3^** | **Beta^4^** | **95% CI^5^** | **p-value^6^** |  | **N^3^** | **Beta^4^** | **95% CI^5^** | **p-value^6^** |
| **CBCL assessed at 2 years** |  |  |  |  |  |  |  |  |  |  |  |  |  |  |
| **Internalizing score** |  |  |  |  |  |  |  |  |  |  |  |  |  |  |
| Specific richness | 332 | 0.07 | -0.13, 0.27 | 0.49 |  | 321 | 0.09 | -0.11, 0.28 | 0.39 |  | 321 | 0.11 | -0.06, 0.28 | 0.19 |
| Shannon diversity | 332 | 0.41 | -0.53, 1.34 | 0.39 |  | 321 | 0.51 | -0.44, 1.46 | 0.29 |  | 321 | 0.52 | -0.43, 1.47 | 0.28 |
| **Externalizing score** |  |  |  |  |  |  |  |  |  |  |  |  |  |  |
| Specific richness | 331 | -0.04 | -0.31, 0.22 | 0.75 |  | 320 | -0.05 | -0.31, 0.22 | 0.74 |  | 320 | 0.01 | -0.22, 0.23 | 0.96 |
| Shannon diversity | 331 | 0.57 | -0.69, 1.82 | 0.37 |  | 320 | 0.54 | -0.74, 1.82 | 0.40 |  | 320 | 0.54 | -0.73, 1.82 | 0.40 |
| **SRS-2 assessed at 3 years** |  |  |  |  |  |  |  |  |  |  |  |  |  |  |
| **Total score** |  |  |  |  |  |  |  |  |  |  |  |  |  |  |
| Specific richness | 327 | 0.21 | -0.32, 0.74 | 0.44 |  | 316 | 0.25 | -0.28, 0.79 | 0.36 |  | 316 | 0.18 | -0.26, 0.63 | 0.42 |
| Shannon diversity | 327 | 0.15 | -2.34, 2.64 | 0.91 |  | 316 | 0.36 | -2.17, 2.89 | 0.78 |  | 316 | 0.35 | -2.17, 2.87 | 0.79 |
| **BRIEF-P assessed at 3 years** |  |  |  |  |  |  |  |  |  |  |  |  |  |  |
| **Inhibition score** |  |  |  |  |  |  |  |  |  |  |  |  |  |  |
| Specific richness | 325 | 0.07 | -0.15, 0.29 | 0.51 |  | 314 | 0.09 | -0.14, 0.31 | 0.45 |  | 314 | 0.04 | -0.14, 0.23 | 0.64 |
| Shannon diversity | 325 | 0.15 | -0.89, 1.19 | 0.78 |  | 314 | 0.30 | -0.76, 1.36 | 0.58 |  | 314 | 0.29 | -0.77, 1.34 | 0.59 |
| **Shift score** |  |  |  |  |  |  |  |  |  |  |  |  |  |  |
| Specific richness | 333 | 0.03 | -0.10, 0.15 | 0.69 |  | 322 | 0.03 | -0.10, 0.16 | 0.64 |  | 322 | 0.04 | -0.07, 0.14 | 0.46 |
| Shannon diversity | 333 | -0.13 | -0.72, 0.46 | 0.67 |  | 322 | -0.03 | -0.63, 0.56 | 0.92 |  | 322 | -0.03 | -0.63, 0.56 | 0.91 |
| **Emotional control score** |  |  |  |  |  |  |  |  |  |  |  |  |  |  |
| Specific richness | 332 | 0.14 | -0.01, 0.28 | 0.06 |  | 321 | 0.15 | 0.00, 0.29 | 0.05 |  | 321 | 0.11 | -0.01, 0.23 | 0.07 |
| Shannon diversity | 332 | 0.55 | -0.14, 1.23 | 0.12 |  | 321 | 0.62 | -0.07, 1.31 | 0.08 |  | 321 | 0.61 | -0.08, 1.31 | 0.08 |
| **Work memory score** |  |  |  |  |  |  |  |  |  |  |  |  |  |  |
| Specific richness | 324 | 0.09 | -0.10, 0.27 | 0.35 |  | 315 | 0.10 | -0.09, 0.28 | 0.32 |  | 315 | 0.06 | -0.10, 0.22 | 0.44 |
| Shannon diversity | 324 | 0.10 | -0.77, 0.98 | 0.81 |  | 315 | 0.16 | -0.72, 1.05 | 0.72 |  | 315 | 0.16 | -0.73, 1.05 | 0.73 |
| **Plan and organization score** |  |  |  |  |  |  |  |  |  |  |  |  |  |  |
| Specific richness | 330 | 0.09 | -0.03, 0.21 | 0.12 |  | 319 | 0.10 | -0.01, 0.22 | 0.08 |  | 319 | 0.08 | -0.02, 0.18 | 0.11 |
| Shannon diversity | 330 | 0.22 | -0.34, 0.77 | 0.44 |  | 319 | 0.25 | -0.31, 0.80 | 0.39 |  | 319 | 0.24 | -0.31, 0.80 | 0.39 |
| **WPPSI-IV assessed at 3 years** |  |  |  |  |  |  |  |  |  |  |  |  |  |  |
| **Verbal comprehension score** |  |  |  |  |  |  |  |  |  |  |  |  |  |  |
| Specific richness | 310 | -0.14 | -0.63, 0.35 | 0.57 |  | 301 | -0.16 | -0.66, 0.33 | 0.52 |  | 301 | -0.13 | -0.54, 0.29 | 0.54 |
| Shannon diversity | 310 | -1.49 | -3.85, 0.88 | 0.22 |  | 301 | -1.41 | -3.81, 1.00 | 0.25 |  | 301 | -1.40 | -3.80, 1.00 | 0.25 |
| **Visuospatial score** |  |  |  |  |  |  |  |  |  |  |  |  |  |  |
| Specific richness | 310 | -0.20 | -0.68, 0.27 | 0.40 |  | 301 | -0.22 | -0.69, 0.24 | 0.35 |  | 301 | -0.13 | -0.52, 0.26 | 0.50 |
| Shannon diversity | 310 | -0.31 | -2.59, 1.96 | 0.79 |  | 301 | -0.43 | -2.69, 1.83 | 0.71 |  | 301 | -0.41 | -2.67, 1.85 | 0.72 |
| **Work memory score** |  |  |  |  |  |  |  |  |  |  |  |  |  |  |
| Specific richness | 310 | -0.10 | -0.57, 0.36 | 0.66 |  | 301 | -0.10 | -0.57, 0.36 | 0.67 |  | 301 | -0.07 | -0.45, 0.32 | 0.74 |
| Shannon diversity | 310 | -1.47 | -3.71, 0.76 | 0.20 |  | 301 | -1.44 | -3.68, 0.80 | 0.21 |  | 301 | -1.42 | -3.65, 0.81 | 0.21 |
| **Total score** |  |  |  |  |  |  |  |  |  |  |  |  |  |  |
| Specific richness | 310 | -0.14 | -0.62, 0.34 | 0.56 |  | 301 | -0.16 | -0.64, 0.32 | 0.50 |  | 301 | -0.11 | -0.51, 0.29 | 0.58 |
| Shannon diversity | 310 | -1.24 | -3.55, 1.07 | 0.29 |  | 301 | -1.27 | -3.59, 1.05 | 0.28 |  | 301 | -1.25 | -3.56, 1.06 | 0.29 |
| ^1^Child Behavior Checklist (CBCL), Social Responsiveness Scale (SRS-2) and Behavior Rating Inventory of Executive Function, Pre-school (BRIEF-P) are continuous raw scores. Wechsler Preschool and Primary Scale of Intelligence (WPPSI-IV) are continuous scores standardized on child age at evaluation and on the neuropsychologist who conducted the test.  ^2^Correspond to the main analysis (Table 2).  ^3^Number of observations.  ^4^Average changes in neurodevelopment scores when the specific richness increased by 10 ASVs or when the Shannon diversity increased by one unit; adjusted for child age at neurodevelopment assessment (except for WPPSI-IV scores that were already standardized), delivery mode, gestational duration, child characteristics (sex, weight and length at birth), breastfeeding duration, period of introduction of solid food, antibiotics use during the first year of life, presence of pets at home, maternal parity, child perinatal passive smoking up to one year, HOME questionnaire total score, main mode of child care at 12 months and maternal characteristics (parity, age and BMI before pregnancy, education, and smoking status during pregnancy, maternal anxiety and depression score during the third trimester of pregnancy).  ^5^CI: Confidence Interval.  ^6^After correction for multiple testing, the significance threshold was 0.002. | | | | | | | | | | | | | | |

Table S6: Sensitivity analysis – Effects of including the HOME covariate (assessed at 3 years) in the models of the estimated effects of the gut microbiota parameters on the CBCL scores assessed at 2 years in the SEPAGES cohort.

| **Gut microbiota parameters** | **Internalizing CBCL score at 2 years** | | | | | | | |  | **Externalizing CBCL score at 2 years** | | | | | | | |
| --- | --- | --- | --- | --- | --- | --- | --- | --- | --- | --- | --- | --- | --- | --- | --- | --- | --- |
|  | **N^2^** | **Adjusted for HOME variable^1^** | | |  | **Not adjusted for HOME variable** | | |  | **N^2^** | **Adjusted for HOME variable^1^** | | |  | **Not adjusted for HOME variable** | | |
|  |  | **Beta^3^** | **95% CI^4^** | **p-value^5^** |  | **Beta^6^** | **95% CI^4^** | **p-value^5^** |  |  | **Beta^3^** | **95% CI^4^** | **p-value^5^** |  | **Beta^6^** | **95% CI^4^** | **p-value^5^** |
| **α-diversity analysis** |  |  |  |  |  |  |  |  |  |  |  |  |  |  |  |  |  |
| Specific richness | 332 | 0.07 | -0.13, 0.27 | 0.49 |  | 0.09 | -0.11, 0.28 | 0.39 |  | 331 | -0.04 | -0.31, 0.22 | 0.75 |  | -0.02 | -0.28, 0.25 | 0.91 |
| Shannon diversity | 332 | 0.41 | -0.53, 1.34 | 0.39 |  | 0.47 | -0.46, 1.41 | 0.32 |  | 331 | 0.57 | -0.69, 1.82 | 0.37 |  | 0.67 | -0.59, 1.93 | 0.29 |
| **Phylum analysis** |  |  |  |  |  |  |  |  |  |  |  |  |  |  |  |  |  |
| Firmicutes | 336 | -0.01 | -0.27, 0.24 | 0.92 |  | 0.01 | -0.25, 0.27 | 0.93 |  | 335 | 0.29 | -0.05, 0.64 | 0.09 |  | 0.33 | -0.01, 0.68 | 0.06 |
| Actinobacteria | 336 | 0.17 | -0.07, 0.40 | 0.16 |  | 0.16 | -0.08, 0.39 | 0.19 |  | 335 | 0.17 | -0.14, 0.49 | 0.28 |  | 0.16 | -0.16, 0.47 | 0.32 |
| Bacteroidetes | 336 | -0.05 | -0.34, 0.24 | 0.73 |  | -0.07 | -0.36, 0.23 | 0.66 |  | 335 | -0.14 | -0.53, 0.25 | 0.48 |  | -0.16 | -0.56, 0.23 | 0.41 |
| Proteobacteria | 336 | -0.28 | -0.60, 0.05 | 0.10 |  | -0.29 | -0.62, 0.04 | 0.09 |  | 335 | -0.48 | -0.92, -0.05 | 0.03 |  | -0.50 | -0.94, -0.06 | 0.03 |
| **Genus analysis** |  |  |  |  |  |  |  |  |  |  |  |  |  |  |  |  |  |
| *Bifidobacterium* | 336 | 0.36 | -0.01, 0.74 | 0.06 |  | 0.37 | -0.01, 0.75 | 0.05 |  | 335 | 0.58 | 0.07, 1.08 | 0.03 |  | 0.59 | 0.08, 1.10 | 0.02 |
| *Bacteroides* | 336 | 0.11 | -0.09, 0.30 | 0.29 |  | 0.10 | -0.10, 0.30 | 0.32 |  | 335 | 0.08 | -0.18, 0.35 | 0.53 |  | 0.07 | -0.19, 0.34 | 0.58 |
| *Blautia* | 336 | 0.02 | -0.18, 0.21 | 0.88 |  | 0.03 | -0.17, 0.22 | 0.79 |  | 335 | 0.17 | -0.09, 0.43 | 0.20 |  | 0.19 | -0.07, 0.45 | 0.16 |
| *Escherichia* and *Shigella* | 336 | -0.04 | -0.24, 0.16 | 0.68 |  | -0.05 | -0.25, 0.15 | 0.66 |  | 335 | -0.05 | -0.31, 0.22 | 0.73 |  | -0.05 | -0.32, 0.22 | 0.70 |
| *Akkermansia* | 336 | 0.06 | -0.07, 0.18 | 0.39 |  | 0.06 | -0.07, 0.19 | 0.36 |  | 335 | 0.05 | -0.12, 0.22 | 0.58 |  | 0.05 | -0.12, 0.22 | 0.54 |
| *Faecalibacterium* | 336 | 0.03 | -0.10, 0.16 | 0.61 |  | 0.03 | -0.10, 0.16 | 0.61 |  | 335 | 0.04 | -0.14, 0.21 | 0.68 |  | 0.04 | -0.14, 0.21 | 0.69 |
| *Streptococcus* | 336 | 0.00 | -0.20, 0.21 | 0.97 |  | 0.01 | -0.19, 0.22 | 0.91 |  | 335 | -0.04 | -0.32, 0.23 | 0.77 |  | -0.03 | -0.31, 0.25 | 0.84 |
| *Clostridium XlVa* | 336 | 0.01 | -0.22, 0.24 | 0.92 |  | 0.00 | -0.23, 0.23 | 0.99 |  | 335 | 0.23 | -0.08, 0.54 | 0.14 |  | 0.22 | -0.09, 0.53 | 0.17 |
| *Lachnospiracea incertae sedis* | 336 | 0.11 | -0.17, 0.39 | 0.44 |  | 0.11 | -0.18, 0.39 | 0.45 |  | 335 | 0.31 | -0.06, 0.69 | 0.10 |  | 0.31 | -0.07, 0.69 | 0.11 |
| *Anaerostipes* | 336 | 0.03 | -0.12, 0.18 | 0.67 |  | 0.03 | -0.12, 0.18 | 0.69 |  | 335 | 0.15 | -0.05, 0.35 | 0.15 |  | 0.15 | -0.06, 0.35 | 0.16 |
| *Gemmiger* | 336 | 0.10 | -0.03, 0.23 | 0.13 |  | 0.11 | -0.02, 0.23 | 0.10 |  | 335 | 0.07 | -0.10, 0.25 | 0.39 |  | 0.09 | -0.08, 0.26 | 0.31 |
| *Ruminococcus* | 336 | 0.01 | -0.11, 0.12 | 0.91 |  | 0.01 | -0.11, 0.12 | 0.92 |  | 335 | 0.04 | -0.12, 0.19 | 0.65 |  | 0.04 | -0.12, 0.19 | 0.65 |
| *Clostridium XVIII* | 336 | 0.21 | -0.10, 0.52 | 0.19 |  | 0.21 | -0.10, 0.52 | 0.18 |  | 335 | 0.52 | 0.11, 0.93 | 0.01 |  | 0.53 | 0.11, 0.94 | 0.01 |
| *Clostridium sensu stricto* | 336 | -0.10 | -0.28, 0.07 | 0.25 |  | -0.09 | -0.27, 0.08 | 0.30 |  | 335 | 0.12 | -0.11, 0.36 | 0.30 |  | 0.14 | -0.10, 0.38 | 0.24 |
| *Enterococcus* | 336 | -0.07 | -0.22, 0.07 | 0.33 |  | -0.07 | -0.22, 0.08 | 0.34 |  | 335 | -0.26 | -0.46, -0.07 | 0.007 |  | -0.26 | -0.46, -0.07 | 0.008 |
| *Cellulosibacter* | 336 | 0.03 | -0.10, 0.16 | 0.65 |  | 0.02 | -0.10, 0.15 | 0.72 |  | 335 | 0.10 | -0.07, 0.27 | 0.25 |  | 0.09 | -0.08, 0.26 | 0.31 |
| *Enterobacter* | 336 | -0.10 | -0.25, 0.06 | 0.22 |  | -0.09 | -0.25, 0.06 | 0.23 |  | 335 | -0.13 | -0.33, 0.08 | 0.22 |  | -0.12 | -0.33, 0.08 | 0.24 |
| *Roseburia* | 336 | 0.05 | -0.09, 0.18 | 0.50 |  | 0.05 | -0.09, 0.18 | 0.47 |  | 335 | 0.08 | -0.10, 0.26 | 0.36 |  | 0.09 | -0.09, 0.27 | 0.34 |
| *Veillonella* | 336 | 0.17 | 0.02, 0.31 | 0.03 |  | 0.14 | -0.01, 0.29 | 0.07 |  | 335 | 0.25 | 0.05, 0.45 | 0.02 |  | 0.21 | 0.01, 0.41 | 0.04 |
| *Fusicatenibacter* | 336 | 0.03 | -0.10, 0.16 | 0.63 |  | 0.03 | -0.10, 0.16 | 0.63 |  | 335 | 0.08 | -0.09, 0.26 | 0.33 |  | 0.08 | -0.09, 0.26 | 0.34 |
| *Parabacteroides* | 336 | 0.16 | 0.02, 0.29 | 0.02 |  | 0.17 | 0.03, 0.30 | 0.02 |  | 335 | 0.05 | -0.14, 0.23 | 0.62 |  | 0.07 | -0.12, 0.25 | 0.47 |
| *Collinsella* | 336 | 0.02 | -0.12, 0.16 | 0.78 |  | 0.03 | -0.11, 0.17 | 0.69 |  | 335 | -0.04 | -0.22, 0.15 | 0.70 |  | -0.02 | -0.21, 0.16 | 0.81 |
| *Alistipes* | 336 | 0.00 | -0.14, 0.13 | 0.97 |  | 0.00 | -0.14, 0.14 | 0.99 |  | 335 | -0.10 | -0.28, 0.09 | 0.30 |  | -0.09 | -0.28, 0.09 | 0.32 |
| *Flavonifractor* | 336 | 0.05 | -0.15, 0.25 | 0.63 |  | 0.04 | -0.16, 0.24 | 0.71 |  | 335 | 0.03 | -0.24, 0.29 | 0.85 |  | 0.01 | -0.26, 0.28 | 0.95 |
| *Ruminococcus 2* | 336 | -0.04 | -0.20, 0.12 | 0.62 |  | -0.02 | -0.18, 0.14 | 0.78 |  | 335 | -0.02 | -0.23, 0.19 | 0.86 |  | 0.01 | -0.21, 0.22 | 0.95 |
| *Clostridium IV* | 336 | 0.03 | -0.14, 0.21 | 0.69 |  | 0.05 | -0.13, 0.22 | 0.61 |  | 335 | -0.01 | -0.24, 0.22 | 0.95 |  | 0.01 | -0.22, 0.24 | 0.93 |
| *Romboutsia* | 336 | 0.04 | -0.12, 0.20 | 0.59 |  | 0.05 | -0.12, 0.21 | 0.57 |  | 335 | 0.10 | -0.11, 0.32 | 0.35 |  | 0.11 | -0.11, 0.32 | 0.34 |
| *Eggerthella* | 336 | 0.07 | -0.13, 0.26 | 0.52 |  | 0.08 | -0.12, 0.28 | 0.42 |  | 335 | 0.12 | -0.14, 0.39 | 0.37 |  | 0.15 | -0.12, 0.41 | 0.28 |
| *Dorea* | 336 | 0.11 | -0.03, 0.25 | 0.13 |  | 0.13 | -0.01, 0.27 | 0.07 |  | 335 | 0.15 | -0.04, 0.34 | 0.12 |  | 0.18 | -0.01, 0.37 | 0.06 |
| *Hungatella* | 336 | 0.13 | -0.02, 0.29 | 0.09 |  | 0.15 | -0.01, 0.30 | 0.06 |  | 335 | 0.13 | -0.08, 0.33 | 0.24 |  | 0.15 | -0.06, 0.36 | 0.16 |
| *Eisenbergiella* | 336 | 0.06 | -0.10, 0.22 | 0.46 |  | 0.06 | -0.09, 0.22 | 0.42 |  | 335 | 0.07 | -0.14, 0.28 | 0.53 |  | 0.08 | -0.14, 0.29 | 0.47 |
| *Klebsiella* | 336 | -0.07 | -0.24, 0.10 | 0.43 |  | -0.06 | -0.23, 0.11 | 0.48 |  | 335 | -0.24 | -0.47, -0.02 | 0.03 |  | -0.23 | -0.46, -0.01 | 0.04 |
| *Intestinibacter* | 336 | 0.05 | -0.13, 0.23 | 0.58 |  | 0.06 | -0.13, 0.24 | 0.53 |  | 335 | 0.05 | -0.19, 0.30 | 0.67 |  | 0.07 | -0.18, 0.31 | 0.60 |
| *Subdoligranulum* | 336 | 0.18 | 0.03, 0.34 | 0.02 |  | 0.19 | 0.04, 0.35 | 0.01 |  | 335 | 0.14 | -0.06, 0.35 | 0.17 |  | 0.16 | -0.05, 0.37 | 0.13 |
| *Dialister* | 336 | 0.00 | -0.18, 0.19 | 0.97 |  | 0.01 | -0.18, 0.19 | 0.95 |  | 335 | 0.00 | -0.25, 0.25 | 0.99 |  | 0.00 | -0.25, 0.25 | 0.99 |
| *Butyricicoccus* | 336 | -0.08 | -0.23, 0.08 | 0.33 |  | -0.08 | -0.23, 0.08 | 0.33 |  | 335 | -0.25 | -0.46, -0.05 | 0.02 |  | -0.25 | -0.46, -0.04 | 0.02 |
| *Erysipelotrichaceae incertae sedis* | 336 | 0.15 | -0.05, 0.34 | 0.14 |  | 0.13 | -0.07, 0.32 | 0.20 |  | 335 | 0.21 | -0.05, 0.47 | 0.11 |  | 0.18 | -0.08, 0.44 | 0.18 |
| *Coprococcus* | 336 | 0.06 | -0.10, 0.23 | 0.46 |  | 0.07 | -0.09, 0.23 | 0.40 |  | 335 | 0.35 | 0.14, 0.57 | 0.002 |  | 0.37 | 0.15, 0.58 | 0.001 |
| *Haemophilus* | 336 | -0.02 | -0.19, 0.15 | 0.79 |  | -0.03 | -0.20, 0.14 | 0.71 |  | 335 | 0.11 | -0.12, 0.33 | 0.36 |  | 0.09 | -0.14, 0.32 | 0.42 |
| *Oscillibacter* | 336 | 0.19 | 0.02, 0.36 | 0.03 |  | 0.19 | 0.02, 0.36 | 0.03 |  | 335 | 0.12 | -0.11, 0.35 | 0.30 |  | 0.13 | -0.10, 0.36 | 0.28 |
| *Terrisporobacter* | 336 | -0.02 | -0.19, 0.16 | 0.86 |  | -0.02 | -0.19, 0.16 | 0.85 |  | 335 | -0.02 | -0.25, 0.21 | 0.85 |  | -0.02 | -0.26, 0.21 | 0.85 |
| *Lactococcus* | 336 | -0.03 | -0.21, 0.16 | 0.78 |  | -0.04 | -0.23, 0.15 | 0.68 |  | 335 | 0.02 | -0.23, 0.27 | 0.86 |  | 0.00 | -0.25, 0.25 | 0.99 |
| *Anaerotruncus* | 336 | 0.14 | -0.07, 0.35 | 0.18 |  | 0.15 | -0.05, 0.36 | 0.15 |  | 335 | 0.02 | -0.26, 0.30 | 0.90 |  | 0.04 | -0.24, 0.32 | 0.79 |
| *Granulicatella* | 336 | -0.19 | -0.42, 0.04 | 0.10 |  | -0.19 | -0.42, 0.04 | 0.10 |  | 335 | -0.16 | -0.47, 0.15 | 0.30 |  | -0.16 | -0.47, 0.15 | 0.31 |
| *Peptoniphilus* | 336 | -0.01 | -0.27, 0.25 | 0.93 |  | 0.00 | -0.26, 0.26 | 0.99 |  | 335 | -0.02 | -0.36, 0.33 | 0.93 |  | 0.00 | -0.35, 0.35 | 0.99 |
| *Saccharibacteria genera incertae sedis* | 336 | -0.01 | -0.27, 0.24 | 0.92 |  | 0.00 | -0.25, 0.26 | >0.99 |  | 335 | 0.13 | -0.21, 0.47 | 0.45 |  | 0.15 | -0.19, 0.50 | 0.38 |
| ^1^Correspond to the main analyses Table 2, Table 4 and Table S4.  ^2^Number of observations.  ^3^Average changes in neurodevelopment scores when the specific richness increased by 10 ASVs or when the Shannon diversity increased by one unit, or when the phylum relative abundance increase by 10%, or when the relative abundance of the genera is multiplied by Euler number e (depending on the explanatory variable considered); adjusted for HOME questionnaire total score assessed at 3 years and child age at neurodevelopment assessment (except for WPPSI-IV scores that were already standardized), delivery mode, gestational duration, child characteristics (sex, weight and length at birth), breastfeeding duration, period of introduction of solid food, antibiotics use during the first year of life, presence of pets at home, maternal parity, child perinatal passive smoking up to one year, main mode of child care at 12 months and maternal characteristics (parity, age and BMI before pregnancy, education, and smoking status during pregnancy, maternal anxiety and depression score during the third trimester of pregnancy).  ^4^CI: Confidence Interval.  ^5^After correction for multiple testing, the significance threshold was 0.002 for diversity analyses and 0.0002 for taxonomy analysis.  ^6^Average changes in neurodevelopment scores when the specific richness increased by 10 ASVs or when the Shannon diversity increased by one unit, or when the phylum relative abundance increased by 10%, or when the relative abundance of the genera was multiplied by Euler number e (depending on the explanatory variable considered); analyses were adjusted for the same covariates as the main analyses but for the HOME questionnaire total score assessed at 3 years, which was not adjusted for in some of the analyses presented here. | | | | | | | | | | | | | | | | | |

Table S7: Sensitivity analysis – Effect of standardization of the WPPSI-IV scores on the neuropsychologist who conducted the test in the models of the estimated effects of the gut microbiota parameters on the WPPSI-IV scores assessed at 3 years in the SEPAGES cohort.

Please refer to the excel file.

Table S8: Sensitivity analysis – Effect of standardization of the gut microbiota parameters on the technical factors (child age at feces collection and MiSeq batch effect) in the models of the estimated effects of the gut microbiota parameters on the neurodevelopmental parameters assessed at two and three years in the SEPAGES cohort.

Please refer to the excel file.

Table S9: Sensitivity analysis – Investigation of non-monotonic associations between gut microbiota parameters and neurodevelopmental parameters assessed at two and three years in the SEPAGES cohort.

Please refer to the excel file.

Figure S1: Directed acyclic graph of the relation between one year child gut microbiota and neurodevelopment at two and three years in the SEPAGES cohort.


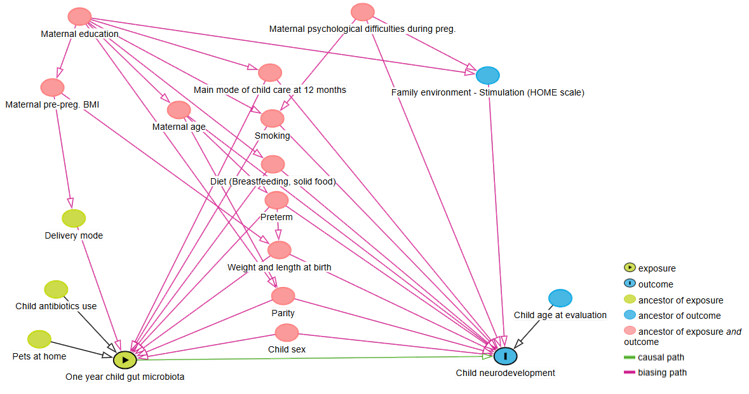
Associations were adjusted for all *a priori* identified ancestors of outcome (in blue), ancestors of predictors (in green) and ancestors of exposures and outcomes (in pink).

Figure S2: Pearson’s correlations between neurodevelopmental parameters (sample sizes between 303 and 335).


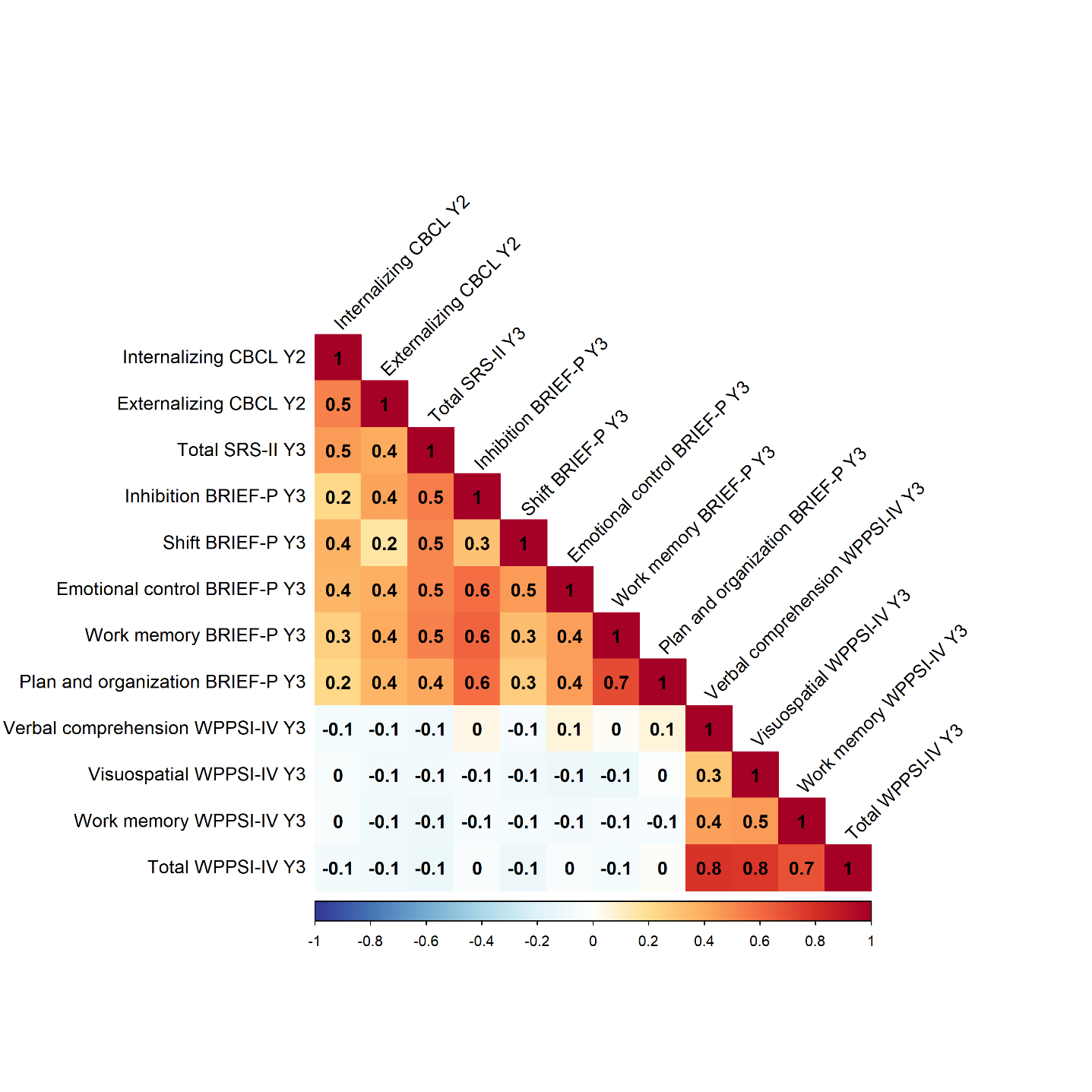


The correlation between each pair of variables was computed using all complete pairs of observations on those variables.

Y2: scores at 2 years, Y3: scores at 3 years.
